# A pressure-based motion intention recognition strategy for phase-specific knee exoskeleton assistance during sit-to-stand

**DOI:** 10.3389/fphys.2026.1859056

**Published:** 2026-09-04

**Authors:** Feng Feng, Yang Yu, Kui Du, Zihao Li, Shuai Chang

**Affiliations:** 1 Department of Physical Education, Capital Normal University, Beijing, China; 2 Sport Biomechanics Center, Capital University of Physical Education and Sports, Beijing, China

**Keywords:** exoskeleton, human–exoskeleton interaction, knee joint moment, motion intention recognition, pressure sensor, sit-to-stand

## Abstract

**Background:**

The sit-to-stand (STS) task is a fundamental functional movement requiring substantial lower-limb joint torque and coordinated neuromuscular control. The knee joint plays a dominant role in generating extensor torque during the body elevation phase. However, existing exoskeleton assistive strategies often lack alignment with the temporal characteristics of joint mechanical demand, limiting their effectiveness.

**Methods:**

Thirteen healthy male participants performed STS tasks under both non-assisted and exoskeleton-assisted conditions. A pressure-based motion intention recognition method was developed using anterior thigh pressure signals to detect movement onset. Three assistive torque levels (3.0 Nm, 4.5 Nm, and 6.0 Nm) were applied to the knee joint. Kinematic and kinetic data were collected using a motion capture system and force plates, and knee joint moment, power, and mechanical work were calculated to evaluate biomechanical changes.

**Results:**

Recognition-performance analysis showed that the pressure-threshold algorithm identified the target pre-extension trigger in all six validation recordings, corresponding to a positive-event detection rate of 100% (6/6). The detected trigger occurred at 0.49 ± 0.05 of the pressure-rising phase, and the onset of assistive torque occurred within 0.072 s of knee-extension onset, advancing torque output by approximately 0.228 s compared with a kinematics-only trigger. Because the validation trials included intentional STS attempts only, specificity and overall classification accuracy against non-intention events were not estimated in the present study. Knee joint moment exhibited a clear phase-dependent pattern, characterized by a rapid increase following seat-off and a peak during the body elevation phase. Compared with the control condition, exoskeleton assistance significantly reduced mean and peak knee joint moment under the 4.5 Nm and 6.0 Nm conditions (p < 0.0167). Joint power and total mechanical work were also significantly reduced across assisted conditions. These reductions were observed in healthy young male participants performing a standardized laboratory STS task.

**Conclusions:**

In healthy young male participants under controlled laboratory conditions, knee joint mechanical demand during STS was strongly phase-dependent, and assistance aligned with this temporal structure reduced knee joint loading. The pressure-based intention recognition method provided a practical approach for improving system responsiveness during this standardized task. However, these findings should be interpreted as preliminary biomechanical evidence obtained from healthy participants, rather than direct evidence of clinical efficacy. Further studies involving older adults, patients with functional impairments, and real-world testing conditions are required before clinical or rehabilitation applications can be inferred.

## 1 Introduction

Lower-limb function plays a fundamental role in maintaining basic mobility, independence, and quality of life. The sit-to-stand (STS) movement, as one of the most essential functional tasks in daily living, represents a typical dynamic transition from a seated to an upright posture, involving coordinated multi-joint movement, neuromuscular activation, and postural control of the lower extremities (Galli et al., 2008). From a physiological perspective, the STS task relies on precise regulation of muscle coordination by the central nervous system, in which feedforward and feedback control mechanisms optimize muscle recruitment and torque generation. This enables rapid forward and upward displacement of the center of mass, while knee extensor torque plays a dominant role in counteracting gravitational load and maintaining stability during the movement (Shum et al., 2005).

However, lower-limb neuromuscular function is susceptible to impairment under various physiological and pathological conditions. For instance, prolonged exposure to microgravity leads to disuse-induced atrophy of lower-limb and trunk muscles, resulting in reduced muscle strength and impaired motor control (Baranowski et al., 2023). Similarly, with the progression of aging, declines in muscle mass and neural drive in older adults are often associated with insufficient torque generation capacity and impaired postural regulation (Hornbrook et al., 1994). Therefore, a systematic investigation of joint torque demands and their underlying neuromuscular control characteristics during the STS task is necessary to identify key determinants of movement performance and to provide a biomechanical basis for assistive strategy design.

Previous studies have analyzed the STS movement from kinematic, kinetic, and electromyographic perspectives, demonstrating that the task exhibits clear phase-dependent characteristics accompanied by dynamic changes in joint torque and muscle activation patterns (Norman-Gerum and McPhee, 2020; Lin and Lee, 2022; Ahn et al., 2024). It has been established that the STS task can be divided into distinct functional phases, each characterized by specific patterns of center-of-mass transfer and mechanical output (Schenkman et al., 1990). Among the lower-limb joints, the knee joint has been identified as the primary contributor to torque generation and a key driver for elevating the body from a seated position (Kerr et al., 1997).

Despite these advances, existing studies have largely focused on descriptive analyses of joint angles, ground reaction forces, and muscle activity, while the intrinsic relationship between joint torque demand and neuromuscular control remains insufficiently understood. From a motor control perspective, successful execution of the STS task depends on precise regulation of activation timing and intensity across multiple muscle groups to ensure a smooth transition between postures. Previous research has highlighted the importance of muscle synergy and neural drive in determining movement efficiency and stability (Hanawa et al., 2017). However, in populations with functional impairments, these control mechanisms are often disrupted, leading to altered activation timing, reduced coordination efficiency, and insufficient torque generation, ultimately compromising movement performance (Papa and Cappozzo, 2000; Hortobágyi et al., 2005). Consequently, analyses based solely on kinematic or kinetic variables may not fully capture the underlying control mechanisms of the STS task. In addition, most studies rely on single-variable approaches, with limited emphasis on systematically characterizing joint torque demand throughout the movement. Therefore, further investigation into the temporal characteristics of joint torque demand is required to identify key limiting factors and to inform assistive strategy development.

In the field of lower-limb assistive technologies, wearable exoskeletons have been widely explored for improving functional movement performance, particularly in tasks such as STS that require substantial knee extensor torque. Previous studies have demonstrated that knee exoskeletons designed for STS assistance are capable of providing joint-level torque output and have shown feasibility in supporting movement under experimental conditions (Shepherd and Rouse, 2017). Studies involving functionally impaired populations further indicate that knee exoskeletons can reduce muscular demand and improve task performance during STS transitions (Sarkisian et al; Gunnell et al., 2025).

Moreover, research on assistance timing suggests that the effectiveness of exoskeleton support is highly dependent on its alignment with the human movement process, as assistance applied at different phases can significantly influence task performance (Choi et al). However, current studies predominantly focus on evaluating assistive performance and control strategies, with limited attention given to the phase-dependent characteristics of actual joint torque demand during the STS task. As a result, assistive torque profiles are often predefined or empirically determined, lacking a solid biomechanical foundation (Balasubramaniam, 2016; Shepherd and Rouse, 2017).

It has been emphasized that the effectiveness of human-exoskeleton interaction is strongly influenced by the consistency between assistive output and the user’s movement demand, whereas mismatched assistance may disrupt movement rhythm and reduce efficiency (Lv et al., 2018). In real-world applications, particularly among frail or elderly individuals, achieving stable and effective assistance remains challenging due to the combined influence of joint torque demand, postural control, and individual variability (Lenzi et al., 2012; Lau and Mombaur, 2024). These findings indicate that assistive effectiveness depends not only on device performance but also on the degree of matching between assistive torque and human joint torque demand. Given that the knee joint plays a dominant role in torque generation during the STS task, and that its demand exhibits a phase-dependent pattern, insufficient understanding of this characteristic may directly limit the effectiveness of assistive strategies.

Given that the knee joint plays a dominant role in torque generation during the STS task, and that its demand exhibits a phase-dependent pattern, insufficient understanding of this characteristic may directly limit the effectiveness of assistive strategies. Accurate and timely motion-intention recognition is therefore essential for phase-specific knee exoskeleton assistance during STS. Several sensing strategies have been used to identify movement initiation, including electromyography (EMG), inertial sensors, joint-angle or angular-velocity thresholds, and external force- or pressure-based detection. EMG signals can provide early information on neuromuscular activation before observable joint motion; however, their application in wearable exoskeleton systems is often limited by electrode placement requirements, signal noise, skin preparation, and sensitivity to environmental and user-dependent factors. Inertial sensors and joint-angle thresholds are easier to integrate into wearable systems, but they mainly detect movement after kinematic changes have already occurred. For powered exoskeletons, this may be too late because communication and actuator response delays can shift assistive torque away from the period of highest mechanical demand. Force plates or seat-pressure systems can detect seat-off events accurately in laboratory settings, but they depend on external environmental instrumentation and are not well suited for portable or daily-use exoskeleton control. In contrast, anterior thigh pressure sensing offers a practical and device-integrated alternative. During STS, contraction of the quadriceps produces soft-tissue expansion beneath the thigh cuff before clear knee extension occurs. A pressure sensor embedded between the anterior thigh and the exoskeleton padding can capture this local mechanical change without requiring skin-mounted electrodes or external laboratory equipment. Compared with posterior thigh or shank pressure signals, anterior thigh pressure showed a more pronounced and consistent rising pattern before knee extension. This signal therefore provides an early and wearable-compatible indicator of STS initiation.

Based on the above considerations, this study aimed to systematically characterize the joint torque demand during the STS task and to utilize these findings to guide the design of an exoskeleton assistance strategy. Healthy male university students were recruited to perform STS movements under controlled conditions. Kinematic and kinetic data were collected to quantify the phase-dependent characteristics of knee joint moment, and to identify critical periods of mechanical demand. Based on these findings, a phase-specific assistance strategy driven by joint torque characteristics was developed and implemented in a knee exoskeleton system, together with an anterior-thigh-pressure-based intention-recognition method designed to improve the temporal synchronization between user movement initiation and assistive torque output. The effectiveness of the proposed strategy was subsequently evaluated through assisted experiments.

It was hypothesized that the knee joint moment during the STS task exhibits a clear phase-dependent pattern, with a peak demand occurring during the body elevation phase. Compared with the non-assisted condition, the proposed assistance strategy is expected to provide better-matched torque support during critical phases, thereby reducing the required biological knee joint moment and altering joint mechanical output during the task.

## 2 Methods

### 2.1 Participants

A total of 13 healthy male university students from Capital University of Physical Education and Sports were recruited for this study. All participants were free from lower-limb musculoskeletal injuries and reported no discomfort during the testing sessions. The mean height and body mass of the participants were 179.7 ± 6.7 cm and 79.8 ± 8.2 kg, respectively. The sample size was determined based on the exploratory and feasibility-oriented nature of this laboratory study. The primary purpose was to characterize phase-dependent knee joint mechanical demand during STS and to conduct an initial within-subject evaluation of the proposed exoskeleton assistance strategy under controlled conditions. No *a priori* power analysis was performed. To reduce inter-individual variability and potential confounding factors related to age, sex, pathology, and neuromuscular impairment, a homogeneous sample of healthy young male participants was recruited for this first-stage validation. All participants completed all experimental conditions, allowing paired within-subject comparisons between the control and assisted conditions. Nevertheless, the small and homogeneous sample limits the generalizability of the findings, and the results should be interpreted as preliminary biomechanical evidence rather than as evidence of clinical efficacy.

All participants provided written informed consent prior to participation. This study was approved by the Ethics Committee of Capital University of Physical Education and Sports (Approval No.: 2025A120) and was conducted in accordance with the Declaration of Helsinki. Prior to the formal experiment, all participants underwent a familiarization session with the exoskeleton device, including instructions on wearing procedures, system operation, and practice of the sit-to-stand task, to ensure consistent and stable performance during data collection.

### 2.2 Exoskeleton system

#### 2.2.1 System structure

**Figure 1:**
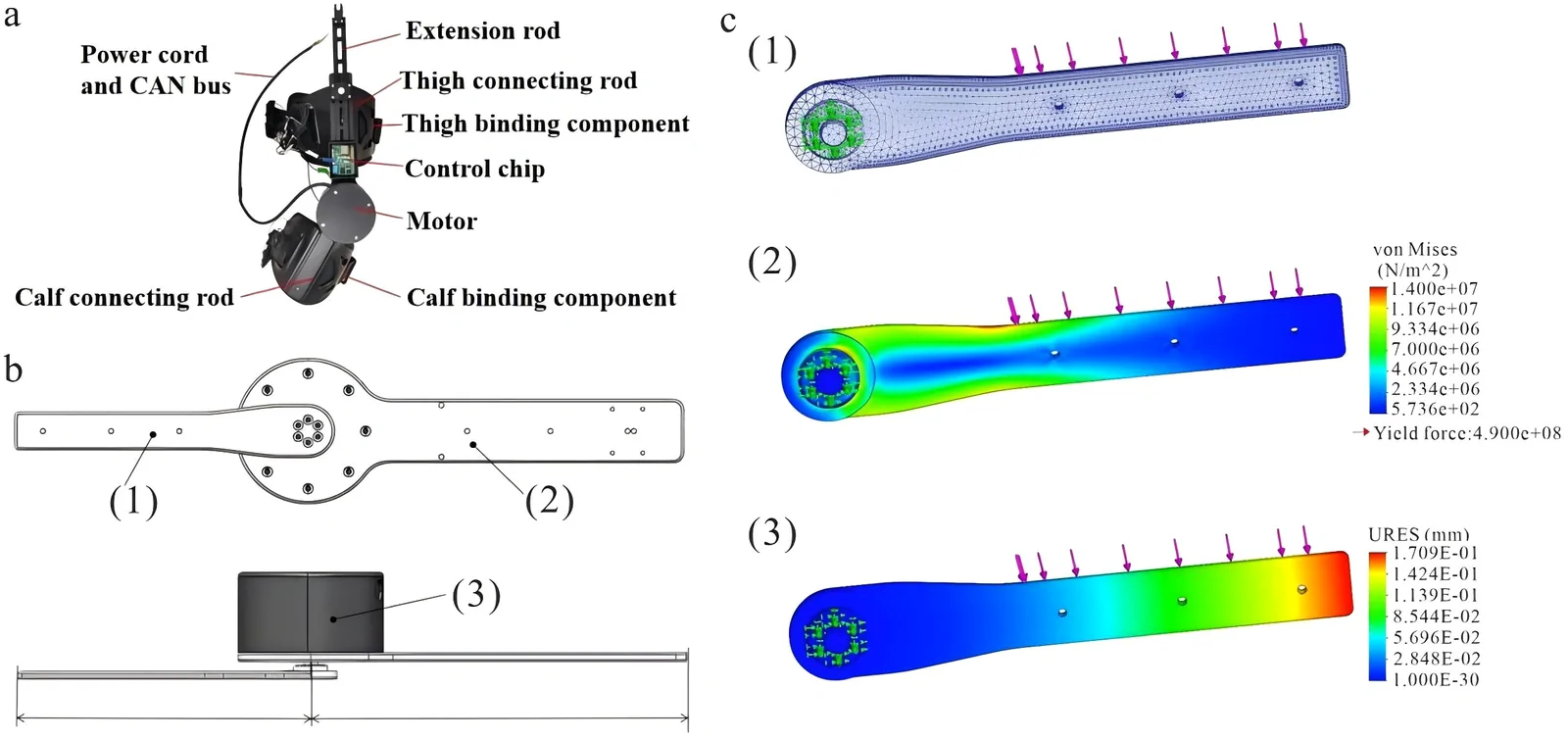
Exoskeleton design. **(A)** Knee exoskeleton prototype; **(B)** linkage mechanism model including (1) thigh link, (2) shank link, and (3) knee joint motor; **(C)** static stress analysis of the shank link, including (1) mesh generation, (2) stress distribution, and (3) deformation results; **(D)** thigh and shank fastening components and their attachment positions, including (1) front view, (2) side view, and (3) rear view; **(E)** fastening system accessories, including **(A)** soft padding, **(B)** dial tensioner, **(C)** waist belt, and **(D)** adjustable strap.

The knee exoskeleton used in this study was designed as a single-degree-of-freedom assistive device, providing flexion-extension torque in the sagittal plane. Considering that knee motion during the sit-to-stand task is primarily dominated by flexion-extension, a fixed-axis linkage mechanism was adopted to ensure stable and controllable torque output, while reducing structural complexity and improving system reliability (**Figure 1**).

**Figure 2:**
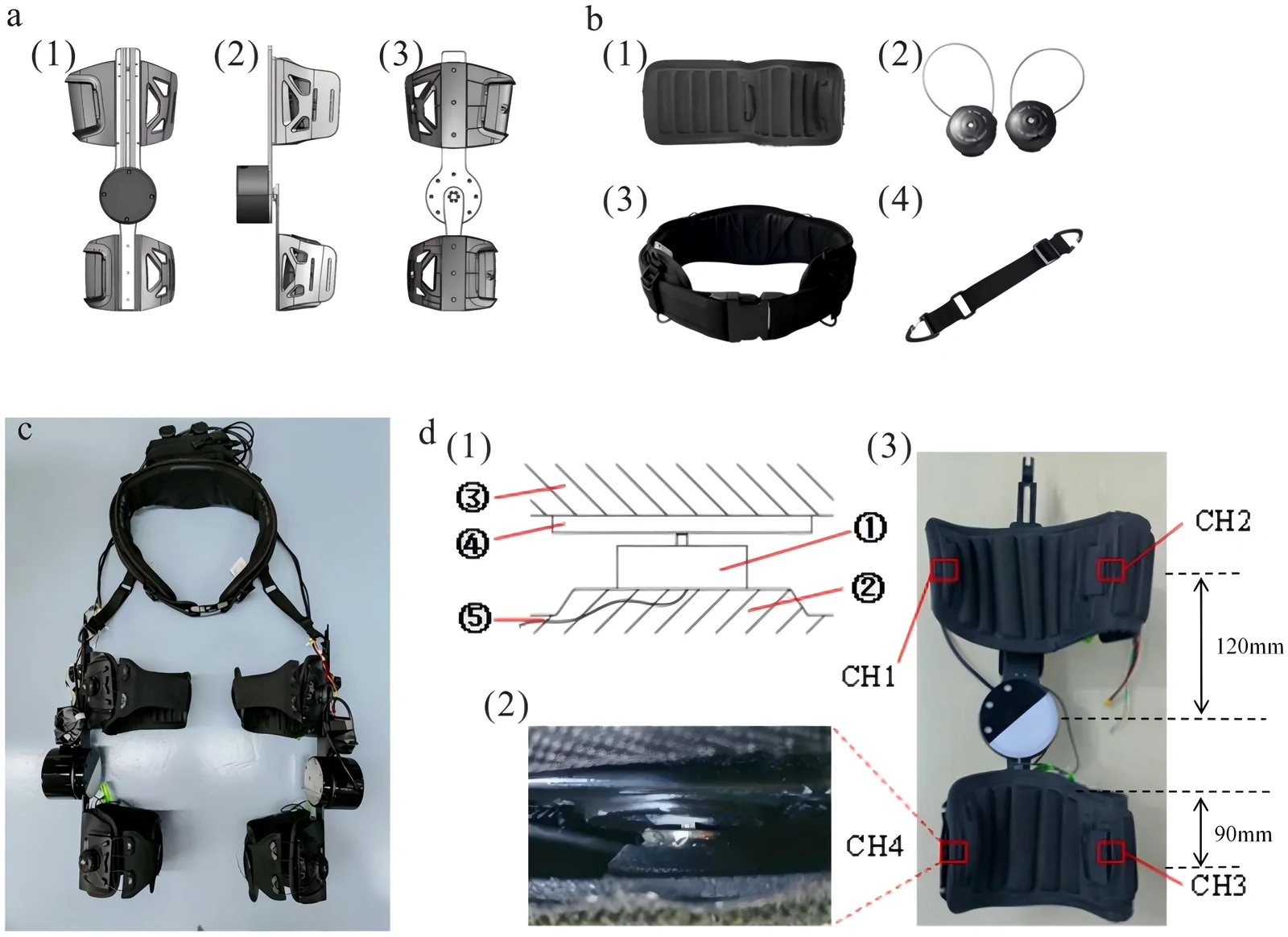
Exoskeleton modification with dimensional annotations for anatomical sensor placement and fastening components. **(A)** Thigh and shank fastening components and their attachment positions, including (1) front view, (2) side view, and (3) rear view; **(B)** fastening system accessories, including (1) soft padding, (2) dial tensioner for quantitative strap preload control, (3) waist belt, and (4) adjustable strap; **(C)** optimized knee exoskeleton prototype with scale marker; **(D)** pressure sensors, including (1) sensor structure and installation, (2) physical sensor, and (3) standardized anatomical sensor placement relative to patellar bony landmarks: CH1 (anterior thigh, 120 mm proximal to the superior pole of the patella), CH2 (posterior thigh, 120 mm proximal to the superior pole of the patella), CH3 (anterior shank, 90 mm distal to the inferior pole of the patella), and CH4 (posterior shank, 90 mm distal to the inferior pole of the patella). Labels: (1) pressure sensor, (2) fastening component, (3) soft padding, (4) insulating layer, (5) signal output wire. CH denotes pressure sensor channel.

The exoskeleton consisted of a thigh link, a shank link, and an actuated knee joint, and was attached to the participant’s thigh and shank using customized fastening components to achieve effective human-exoskeleton coupling. To enhance wearing stability and human-machine interaction comfort, fastening structures were positioned at the mid-thigh and mid-shank regions, incorporating soft padding and adjustable elements to optimize pressure distribution and reduce relative motion during movement (**Figure 2**).

**Figure 3:**
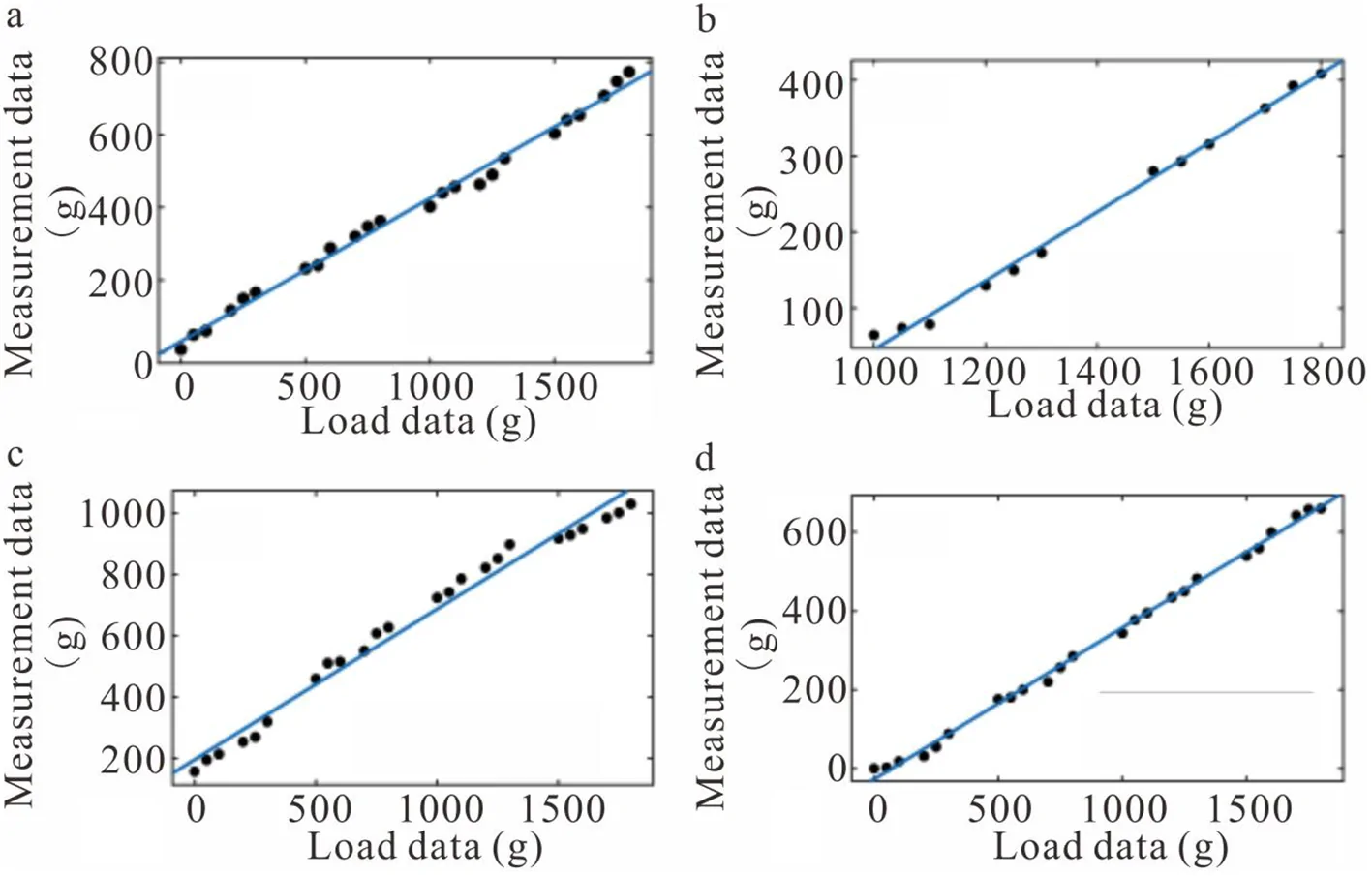
Calibration curves of pressure sensors. **(A)** CH1, **(B)** CH2, **(C)** CH3, and **(D)** CH4.

Assistive torque was generated by the joint motor, with adjustable output levels under different control conditions. To quantify the interaction forces between the exoskeleton and the human body, pressure sensors were embedded at standardized anatomical attachment locations with precise distance markers relative to bony landmarks (**Figure 2D(3)**), and calibrated prior to testing, demonstrating a strong linear relationship between sensor output and applied load (**Figure 3**).

Detailed anatomical placement specifications for four pressure sensor channels (CH1–CH4) were defined as follows:

1. CH1 (anterior thigh pressure sensor): Centered at the mid-thigh sagittal plane, 120 mm proximally upward from the superior pole of the patella; the sensor’s geometric center aligns with the rectus femoris muscle belly at mid-thigh.
2. CH2 (posterior thigh pressure sensor): Symmetrically placed on the posterior mid-thigh, 120 mm proximal to the patella superior pole, matching the vertical height of CH1.
3. CH3 (anterior shank pressure sensor): Mounted 90 mm distal to the inferior pole of the patella on the anterior tibial surface, corresponding to the mid-shank region.
4. CH4 (posterior shank pressure sensor): Located at the posterior mid-shank, 90 mm distal to the patella inferior pole, horizontally aligned with CH3.

All soft padding and sensor units were fixed to customized fastening cuffs. Strap preload and tightness were quantitatively standardized via dial tensioners integrated on each fastening strap (**Figure 2B(2)**). Before each trial, all straps were uniformly tightened to a tension of 15 aN (calibrated via a portable digital tension gauge) to maintain consistent initial preload between the sensor, soft padding, and limb skin surface. After tightening, we recorded the static baseline pressure signal of each channel for 3 s to confirm stable preload without abnormal offset; participants re-donned the exoskeleton if baseline pressure drift exceeded ±5 kPa to eliminate inter-trial preload variability.

**Figure 4:**
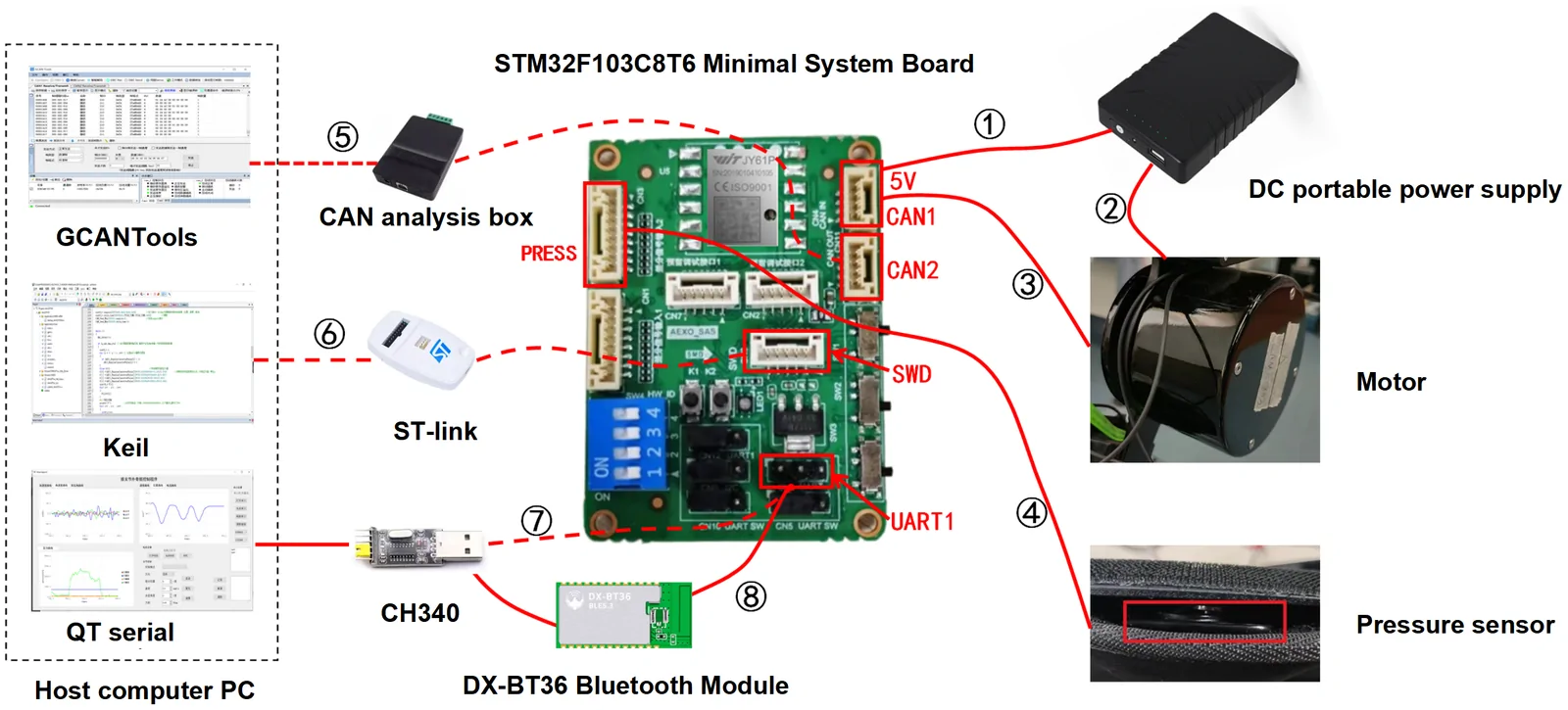
Hardware architecture of the exoskeleton system. The connections labeled(1)-(8) in this image illustrate the relationships among the hardware components of the knee exoskeleton system. Specifically, the CAN interfaces were used for communication between the control board and external devices, as well as for power supply to the system. The joint motor was powered by an external DC source and communicated with the control board via the CAN bus. Pressure sensors were powered by the control board and connected through analog input interfaces, where the acquired signals were converted into digital data. During system development and debugging, auxiliary devices such as a CAN analyzer, programmer, and serial communication module were temporarily connected to the control board for data monitoring, program uploading, and communication with the host computer. For wireless operation, a Bluetooth module was used to enable real-time data transmission between the control board and the host computer.

The overall system comprised an actuation unit, a sensing module, and a control unit. These components were coordinated through an embedded control system to enable real-time data communication and ensure stable and responsive assistive torque output (**Figure 4**). Based on the hardware configuration described above, a control strategy was developed to enable real-time assistance during the sit-to-stand task. The human-machine interaction interface is provided in the Supplementary Materials (**Supplementary Figure 1**), while the motion-intention recognition algorithm used for real-time control is described below.

#### 2.2.2 Pressure-based motion intention recognition algorithm

The pressure-based motion intention recognition algorithm was implemented using the anterior thigh pressure channel (CH1), which showed the most pronounced pressure increase before knee extension during STS. The pressure sensing module operated at a raw sampling frequency of 67 Hz, corresponding to a sampling interval of approximately 15 ms. Before each STS trial, participants remained in a static seated posture for 3 s, and the baseline anterior thigh pressure was calculated as:


$$P_{base}=\frac{1}{N_{base}}\sum_{i=1}^{N_{base}}P_{raw}\left(i\right)$$

Where: P_base_: Static baseline pressure of CH1 (unit: g); N_base_: Total sampling points within 3 s static window, N_base_ = 200; P_raw_(i): Original calibrated pressure value of the i-th sampling point after sensor linear fitting calibration.

During real-time operation, raw pressure data were first smoothed using a moving average filter with a window length of 10 consecutive sampling points:


$$P_{avg}\left(k\right)=\frac{1}{W_{smooth}}\sum_{t=k-W_{smooth+1}}^{k}P_{raw}\left(j\right)$$

where W_smooth_ = 10, and P_avg_ (k) is the smoothed anterior thigh pressure at time index k. To detect the rising trend of anterior thigh pressure, two consecutive judgment windows were compared. Each judgment window contained five smoothed pressure values:


$$A_{new}=\frac{1}{W_{judge}}\sum_{m=0}^{W_{judge}-1}P_{avg}\left(k-m\right)$$

$$A_{old}=\frac{1}{W_{judge}}\sum_{m=W_{judge}}^{2W_{judge}-1}P_{avg}\left(k-m\right)$$

where W_judge_ = 5. Therefore, each real-time judgment cycle incorporated 10×5 = 50 raw sampling points, corresponding to a time window of approximately 0.75 s. A preliminary pressure-rising event was identified when:


$$A_{new}>A_{old}+\Delta P_{thresh}$$

Where ΔP_thresh_ = 50 ag was used to suppress small pressure fluctuations caused by limb vibration or strap micro-slippage.

After the pressure-rising condition was satisfied, the algorithm evaluated whether the smoothed anterior thigh pressure reached the individualized activation threshold:


$${\text{P}}_{\text{trigger}}={\text{C}}_{\text{thresh}}\times {\text{P}}_{\text{base}}$$

Where C_thresh_ = 1.89. This coefficient corresponded to the midpoint of the normalized anterior thigh pressure-rising phase derived from offline analysis. When P_avg_(k)≥P_trigger_, the system generated an STS motion-intention signal and activated knee assistive torque output according to the assigned assistance condition:


$$I_{STS}\left(k\right)=\{\begin{array}{c} 1,A_{new}\left(k\right)-A_{old}\left(k\right)\geq \Delta P_{thresh}\;and\;P_{avg}\left(k\right)\geq P_{trigger}\\ 0,otherwise\\ \tau_{assist}\left(k\right)=I_{STS}\left(k\right)\times \tau_{level} \end{array}$$

where I_STS_(k) is the binary intention-recognition output and τ_level_ represents the assigned peak assistive torque level, namely 3.0 Nm, 4.5 Nm, or 6.0 Nm.

The temporal performance of the recognition algorithm was quantified by comparing the intention-trigger time with the knee-extension onset obtained from motion-capture kinematics:


$${\text{T}}_{\text{delay}}={\text{T}}_{\text{knee\_ext}}-{\text{T}}_{\text{intention}}$$

where T_intention_ is the time at which the pressure-based intention signal was generated, and T_knee_ext_ is the onset of knee extension defined from knee angular velocity. A positive value indicates that the pressure-based trigger occurred before or around the mechanical onset of knee extension.

Algorithm pseudocode:

Input: Raw anterior thigh pressure signal P_raw_, baseline duration 3 s, W_smooth_=10, W_judge_=5, ΔP_thresh_=50 g, C_thresh_=1.89, assigned assistive torque level τ_level_.

Output: STS intention signal I_STS_ and assistive torque command τ_assist_.

1. Record anterior thigh pressure during a 3 s static seated baseline.
2. Calculate baseline pressure P_base_.
3. Set individualized trigger threshold P_trigger_=1.89×P_base_.
4. Continuously acquire anterior thigh pressure at 67 Hz.
5. Smooth raw pressure using a 10-point moving average window.
6. Calculate A_new_ and A_old_ from two consecutive 5-point judgment windows.
7. If A_new_−A_old_≥50 g, identify a preliminary pressure-rising event.
8. If P_avg_≥P_trigger_, set I_STS_=1.
9. Activate assistive torque command: τ_assist_=I_STS_×τ_level_.
10. Otherwise, set I_STS_=0 and τ_assist_=0.
11. Calculate recognition timing using T_delay_=T_knee_ext_-T_intention_.

#### 2.2.3 Recognition-performance evaluation

To provide quantitative performance metrics for the pressure-based intention-recognition strategy, the algorithm was evaluated using six additional exoskeleton-wearing STS recordings. Knee-extension onset, defined by the onset of positive knee angular velocity, was used as the biomechanical reference event. In the present study, “true movement onset” for evaluating recognition timing was operationally defined as knee-extension onset derived from motion-capture kinematics. Specifically, the knee joint angle trajectory was obtained from the motion-capture system, and knee angular velocity was calculated as the first derivative of the knee angle. The reference onset time was identified as the time point at which knee angular velocity changed from non-positive to positive, indicating the initiation of active knee extension during STS. Force-plate data were used for kinetic analysis and phase characterization, but not as the primary reference for intention-recognition timing. EMG activation was also not used as the reference event because EMG onset reflects neuromuscular preparation rather than the mechanical onset of joint motion.

A successful positive-event detection was defined as an intention trigger generated during the rising phase of the anterior-thigh pressure signal before or around the onset of knee extension. The positive-event detection rate was calculated as the number of correctly detected intentional STS events divided by the total number of validation STS recordings. Timing performance was quantified as the temporal offset between assistive-torque onset and knee-extension onset, and detection consistency was assessed using the relative quantile location of the detected trigger within the pressure-rising phase. Because the validation experiment consisted only of intentional STS attempts and did not include non-intention or negative trials, specificity, false-positive rate, and overall classification accuracy were not calculated.

#### 2.2.4 Assistive torque control and torque-tracking evaluation

The three assistance levels used in this study, namely 3.0 Nm, 4.5 Nm, and 6.0 Nm, refer to the commanded peak assistive torque levels. The assistive control framework consisted of a pressure-threshold-based high-level intention-recognition layer and a low-level motor torque-control layer. The high-level controller was updated at 67 Hz, corresponding to the sampling frequency of the pressure-sensing module. Once the STS intention signal was detected, the commanded assistive torque was generated according to:


$$\tau_{cmd}\left(t\right)=I_{STS}\left(t\right)\times \tau_{level}$$

where τ_cmd_(t) is the commanded assistive torque, I_STS_(t) is the binary intention-recognition output, and τ_level_ is the assigned commanded peak torque level. Torque commands were transmitted to the embedded motor controller through the CAN communication interface. The low-level actuator was operated in torque-control mode using the embedded motor controller, with the command update frequency set at 50 Hz.

To distinguish commanded torque from actuator output, motor-output feedback was analyzed during assisted trials. The measured assistive torque τ_meas_(t) was obtained from motor-driver torque feedback. Commanded torque, measured torque, anterior thigh pressure, knee-extension onset, and seat-off timing were aligned during post-processing according to the synchronization procedure described above.

Torque-tracking error was calculated as:


$$e_{\tau }\left(t\right)=\tau_{cmd}\left(t\right)\times \tau_{means}\left(t\right)$$

Tracking performance was quantified using root-mean-square error, mean absolute error, and peak absolute error:


$$RMSE_{\tau }=\frac{1}{N}\sum_{i=1}^{N}e_{\tau }{\left(i\right)}^{2}$$

$$MAE_{\tau }=\frac{1}{N}\sum_{i=1}^{N}\left|e_{\tau }\left(i\right)\right|$$

$$Peak\;Error_{\tau }=max\left|e_{\tau }\left(i\right)\right|$$

where N is the number of samples within the active-assistance interval. Representative torque-time traces and torque-tracking metrics were used to verify whether the actuator output followed the commanded torque profiles under the 3.0 Nm, 4.5 Nm, and 6.0 Nm assistance conditions.

### 2.3 Experimental equipment

Kinematic data were collected using an eight-camera motion capture system (OptiTrack, NaturalPoint, USA) at a sampling frequency of 200 Hz. Ground reaction force (GRF) data were recorded using a force platform (Kistler, Switzerland) at 1,000 Hz. Surface electromyography (sEMG) signals were acquired using a wireless EMG system (Delsys Trigno, Delsys Inc., USA) at a sampling frequency of 2,000 Hz. Bipolar electrodes were placed on five lower-limb muscles of the dominant leg, including the rectus femoris (RF), vastus medialis (VM), vastus lateralis (VL), lateral gastrocnemius (GL), and tibialis anterior (TA), following standard SENIAM recommendations. All measurement systems (motion capture, force plate, and EMG) were synchronized via a hardware trigger to ensure temporal alignment of kinematic, kinetic, and muscle activity data. However, the exoskeleton embedded control system, including the pressure sensors, joint encoder, and motor-output signals, operated through an independent acquisition and control loop and was not directly connected to the same hardware trigger. Therefore, exoskeleton-derived signals were recorded separately and subsequently aligned with the hardware-synchronized biomechanical data during post-processing. A custom knee exoskeleton was used to provide assistive torque during the sit-to-stand task. The device consisted of a single-degree-of-freedom mechanism acting in the sagittal plane, with adjustable assistive torque levels.

### 2.4 Experimental procedure

#### 2.4.1 Sit-to-stand baseline experiment

**Figure 5:**
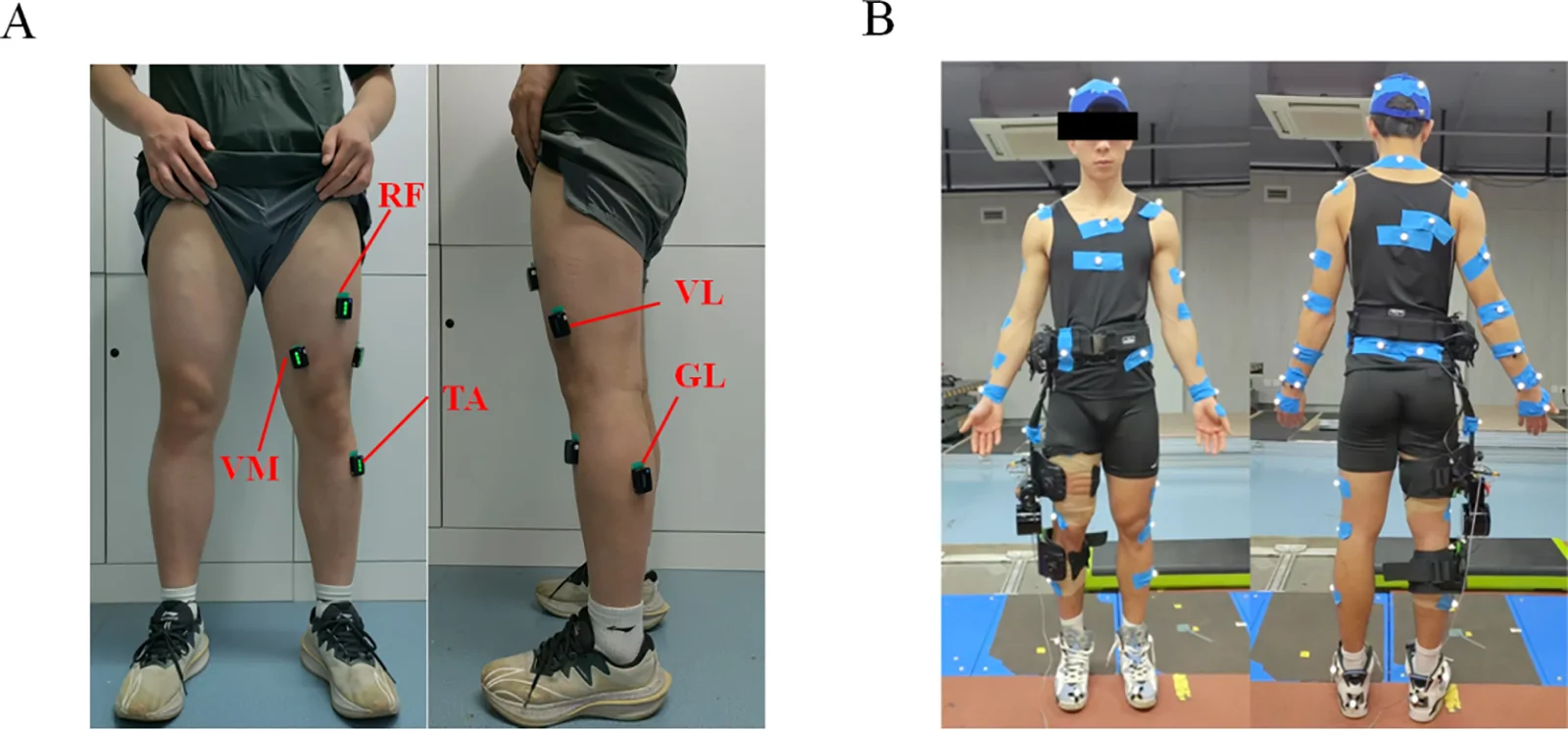
Experimental setup for signal acquisition. **(A)** Placement of surface electromyography (EMG) electrodes on the rectus femoris (RF), vastus medialis (VM), vastus lateralis (VL), gastrocnemius lateralis (GL), and tibialis anterior (TA); **(B)** configuration of reflective markers for the motion capture system, including 39 anatomical landmarks used for three-dimensional kinematic reconstruction.

**Figure 6:**
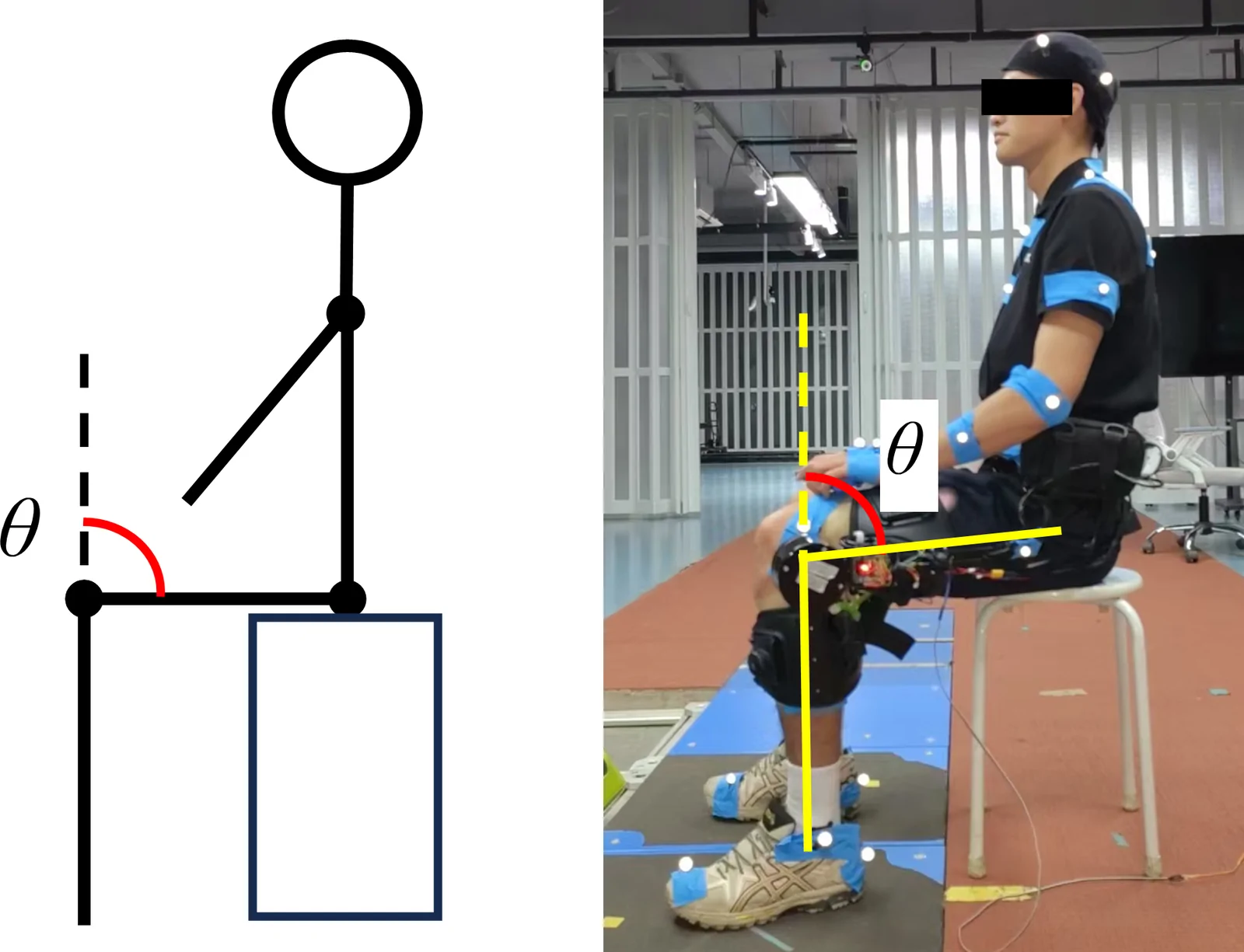
Definition of the knee joint angle (Liu, 2004). During the experiment, the initial posture was controlled by adjusting the exoskeleton structure to set the knee joint angle at 80°, 85°, 90°, 95°, and 100°, while all other experimental conditions were kept consistent. Participants stood with both feet placed on the force plates and arms relaxed at their sides, and performed the sit-to-stand movement following standardized verbal instructions.

Participants performed standardized sit-to-stand movements without exoskeleton assistance to characterize the natural biomechanical patterns. Prior to testing, surface electromyography (EMG) electrodes and reflective markers were placed according to the predefined protocol, as illustrated in **Figure 5** The definition of the knee joint angle is presented in **Figure 6**.

**Figure 7:**
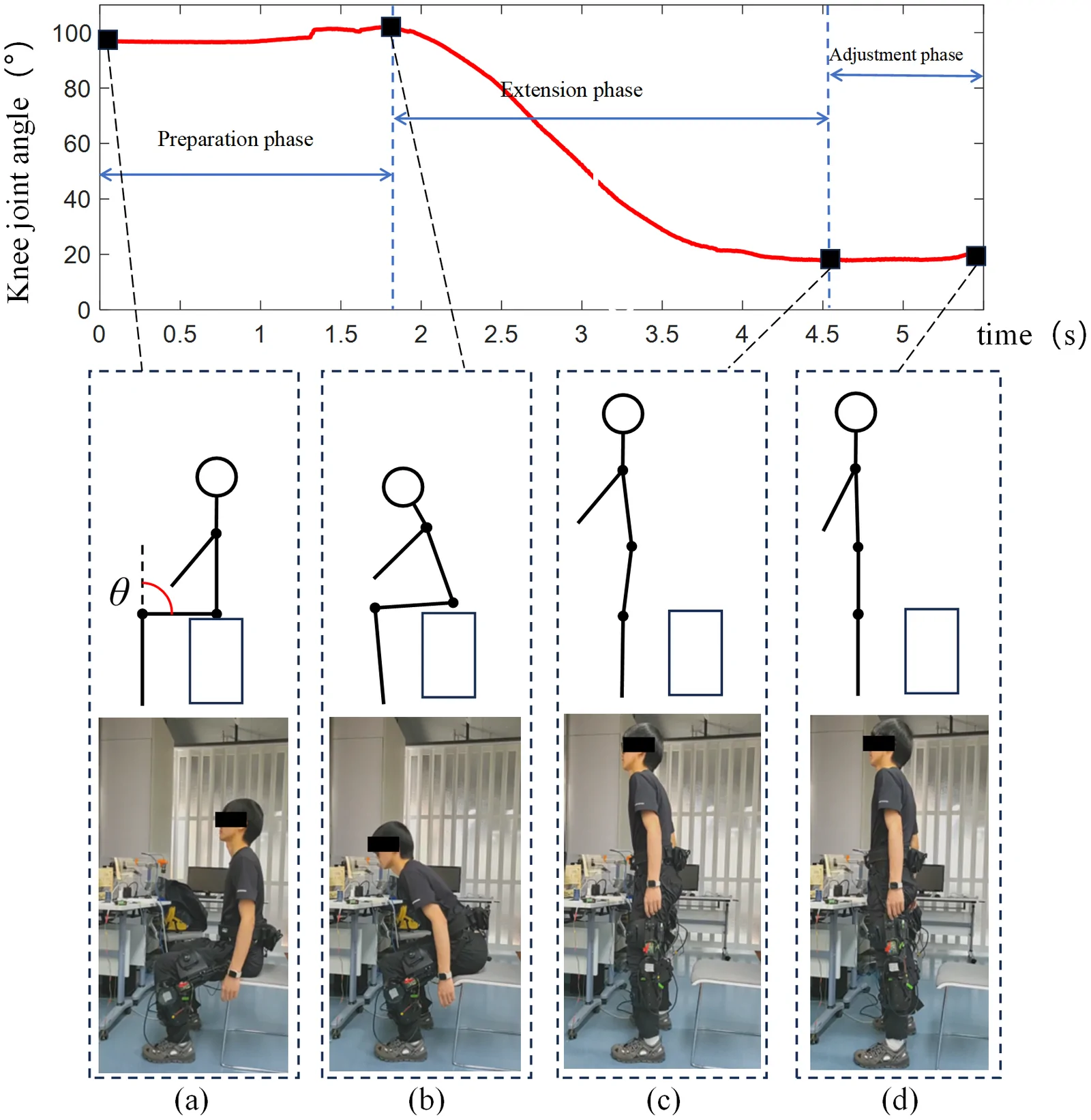
Sit-to-stand movement cycle. **(A)** seated posture; **(B)** seat-off; **(C)** stabilization of the knee joint angle; **(D)** stable standing.

The order of the five initial knee-angle conditions (80°, 85°, 90°, 95°, and 100°) was randomized for each participant using a computer-generated random sequence. The randomization was performed independently for each participant to avoid a systematic order effect. Each condition was repeated three times, with a 30 s rest interval between trials to minimize fatigue and learning effects. Because each STS trial was brief and participants were healthy young adults, this rest interval was considered sufficient to reduce short-term fatigue during the standardized baseline task. All signals were synchronously acquired a hardware-triggered system. To ensure comparability across trials, the movement cycle was defined based on knee joint angle variation, and phase segmentation was conducted according to the criteria shown in **Figure 7**. As shown in **Figure 7**, the sit-to-stand movement was characterized by four key events: seated posture, seat-off, stabilization of the knee joint angle, and stable standing. Based on the knee joint angle trajectory and movement characteristics, the movement was further divided into three phases: preparation, extension, and stabilization. The preparation phase spans from seated posture to seat-off, during which the knee and hip joints gradually flex and the center of mass moves forward and downward. The extension phase extends from seat-off to the stabilization of the knee joint angle, characterized by continuous knee extension and decreasing joint angle accompanied by hip extension. The stabilization phase spans from knee angle stabilization to stable standing, during which the knee joint angle remains relatively constant while the body undergoes minor adjustments to maintain balance.

#### 2.4.2 Exoskeleton-assisted experiment

Following the baseline experiment, participants performed the sit-to-stand task while wearing the knee exoskeleton under assisted conditions. Four experimental modes were tested: a control condition without exoskeleton assistance (Control) and three assistance levels corresponding to peak assistive torques of 3.0 Nm, 4.5 Nm, and 6.0 Nm.

Prior to data collection, the exoskeleton was individually adjusted to ensure proper fit and alignment between the mechanical joint axis and the anatomical knee joint axis. Participants were given sufficient time to familiarize themselves with the device and practice the movement to minimize potential learning effects.

During each trial, participants were instructed to adopt a standardized initial posture identical to that used in the baseline experiment, with both feet placed on the force plates and arms relaxed at their sides. Upon a verbal cue, participants initiated the sit-to-stand movement at a self-selected, natural speed. The exoskeleton provided assistive torque in the knee flexion-extension direction throughout the movement.

The order of the four assistance conditions (Control, 3.0 Nm, 4.5 Nm, and 6.0 Nm) was randomized for each participant using a computer-generated random sequence. This randomization was conducted independently for each participant to reduce systematic order effects. Each condition was repeated three times, with a 30 s rest interval between trials to minimize fatigue and learning effects. The 30 s interval was selected because each STS trial was short in duration, the task was performed at a self-selected natural speed, and the participants were healthy young male adults who reported no discomfort during testing. Nevertheless, the condition order was randomized but not formally counterbalanced across participants. Kinematic, kinetic, and EMG data were acquired using the hardware-triggered laboratory measurement system. Exoskeleton sensor and motor data were acquired independently by the embedded control system and were later synchronized with the laboratory biomechanical data during post-processing.

To ensure temporal consistency between the independently recorded exoskeleton data and the hardware-synchronized laboratory data, *post hoc* alignment was performed using characteristic features of the knee joint angle trajectory. Specifically, the peak knee joint angle associated with the backward foot-adjustment movement was used as a common reference event to estimate the temporal offset between the exoskeleton encoder signal and the motion-capture-derived knee angle. This *post hoc* alignment was applied only to exoskeleton-derived signals; no additional alignment was required among the motion capture, force plate, and EMG data because these systems were hardware synchronized. All data were subsequently time-normalized to 0–100% of the movement cycle according to the phase definition described in **Figure 7**.

### 2.5 Data processing

The EMG signals were processed using a second-order Butterworth band-pass filter (20–300 Hz) to remove motion artifacts and high-frequency noise, followed by a 50 Hz notch filter to eliminate power line interference. The signals were then full-wave rectified and smoothed to obtain the linear envelope representing muscle activation. All EMG data were time-normalized to the movement cycle and amplitude-normalized to reduce inter-subject variability. Kinematic and ground reaction force (GRF) data were preprocessed in Motive before musculoskeletal modeling. For each trial, the data were trimmed from 0.5 s before seat-off to 0.2 s after stable standing to retain the complete STS movement while removing unnecessary static periods. Missing marker trajectories were inspected visually and reconstructed using spline interpolation when short gaps occurred due to temporary marker occlusion. Trials with marker loss that could not be reliably reconstructed were rechecked and corrected before export. Marker trajectories were filtered using a second-order Butterworth low-pass filter with a cut-off frequency of 6 Hz. The processed motion-capture and GRF data were exported in.c3d format and converted into OpenSim-compatible.trc marker trajectory files and.mot GRF files. To reduce computational complexity in inverse dynamics, the center of pressure was fixed using the heel marker location, consistent with the preprocessing procedure used in the original biomechanical analysis. A subject-specific musculoskeletal model was developed in OpenSim 4.4 using a 39-marker full-body lumbar spine (FBLS) model. Joint kinematics and kinetics were computed using inverse kinematics and inverse dynamics, and muscle forces were estimated using static optimization. During model scaling, the root mean square (RMS) marker error was controlled below 0.03 m to ensure model accuracy. Under exoskeleton-assisted conditions, the assistive torque generated by the device was incorporated into the model as an external joint moment to represent human-exoskeleton interaction.

### 2.6 Statistical analysis

All data are presented as mean ± standard deviation (Mean ± SD). For descriptive results, including the time-varying characteristics of knee joint angle, knee joint moment, muscle activation, and muscle force during the sit-to-stand task, only waveform description and visualization were performed. For the exoskeleton-assisted experiment, variables including mean joint moment, peak joint moment, mean joint power, peak joint power, and joint work were first tested for normality, and all variables were normally distributed. Paired-sample t-tests were then used to compare each assistance condition with the control condition. Since three pairwise comparisons were conducted for each variable (Control vs. 3.0 Nm, 4.5 Nm, and 6.0 Nm), Bonferroni correction was applied to control Type I error, and the adjusted significance level was set at p<0.0167. All statistical analyses were performed using SPSS 25.0 (IBM Corp., Armonk, NY, USA). Because no *a priori* power analysis was conducted, the statistical findings should be interpreted cautiously and considered supportive of preliminary biomechanical efficacy within the tested healthy cohort.

## 3 Results

### 3.1 Knee joint biomechanics during sit-to-stand

**Figure 8:**
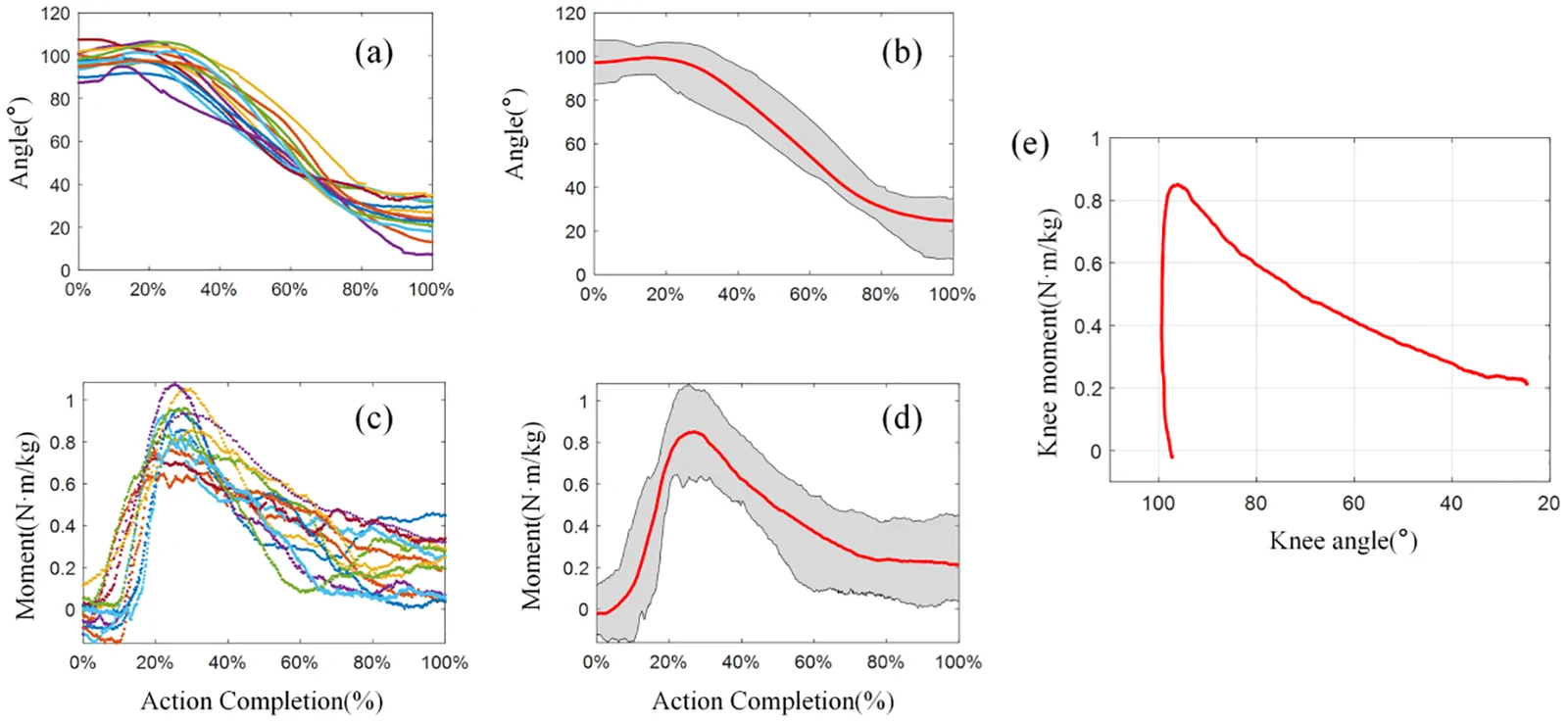
| Knee joint angle, knee joint moment, and knee moment–angle relationship during sit-to-stand from an initial knee flexion angle of 90° in 13 participants. **(A)** Individual knee joint angle trajectories; **(B)** knee joint angle envelope and mean curve; **(C)** individual knee joint moment trajectories; **(D)** knee joint moment envelope and mean curve; and **(E)** knee joint moment–angle curve during sit-to-stand from an initial knee flexion angle of 90°. The gray shaded areas in panels **(B, D)** represent mean ± SD. Knee angle is expressed in degrees (°), and knee moment is normalized to body mass (N·m/kg). Note that a brief knee flexion commonly occurred at the initial phase of sit-to-stand, resulting in a multi-valued relationship in the knee moment–angle curve..

The knee joint angle decreased progressively throughout the sit-to-stand movement, ranging from 97.2° to 24.7°. A slight flexion was observed in the early phase, reaching a peak angle of 99.5° at approximately 15% of the movement cycle, followed by continuous extension until standing (**Figure 8A**). The knee joint moment exhibited a characteristic increase-decrease pattern, with a peak value of approximately 0.85 Nm/kg occurring at around 27% of the movement cycle, after which it gradually decreased and stabilized at approximately 0.20 Nm/kg in the standing phase (**Figure 8B**). The mean trends with standard deviation further confirmed consistent patterns across participants for both knee joint angle and moment (**Figures 8C, D**). In addition, the moment-angle relationship revealed a nonlinear pattern, with joint moment increasing rapidly within a small angular range after seat-off, indicating that the early extension phase represents the period of highest mechanical demand during the sit-to-stand task (**Figure 8E**).

### 3.2 Recognition performance of the pressure-based intention-recognition strategy

The pressure-based intention-recognition algorithm identified the target pre-extension trigger in all six validation recordings, corresponding to a positive-event detection rate of 100% (6/6). The detected trigger locations occurred at 0.52, 0.51, 0.55, 0.50, 0.44, and 0.42 of the pressure-rising phase, with a mean value of 0.49 ± 0.05 (Supplementary2). The onset of assistive torque occurred within 0.072 s of the motion-capture-defined knee-extension onset, which was determined from the positive zero-crossing of knee angular velocity. Compared with a strategy based solely on kinematic data, the pressure-based method advanced the timing of torque output by approximately 0.228 s. These findings indicate that the pressure-threshold strategy provided reliable early detection of intentional STS initiation and improved temporal synchronization between user movement and exoskeleton assistance. Since no non-intention trials were included, specificity and overall classification accuracy were not estimated in the present study.

### 3.3 Assistive torque output and tracking performance

**Figure 9:**
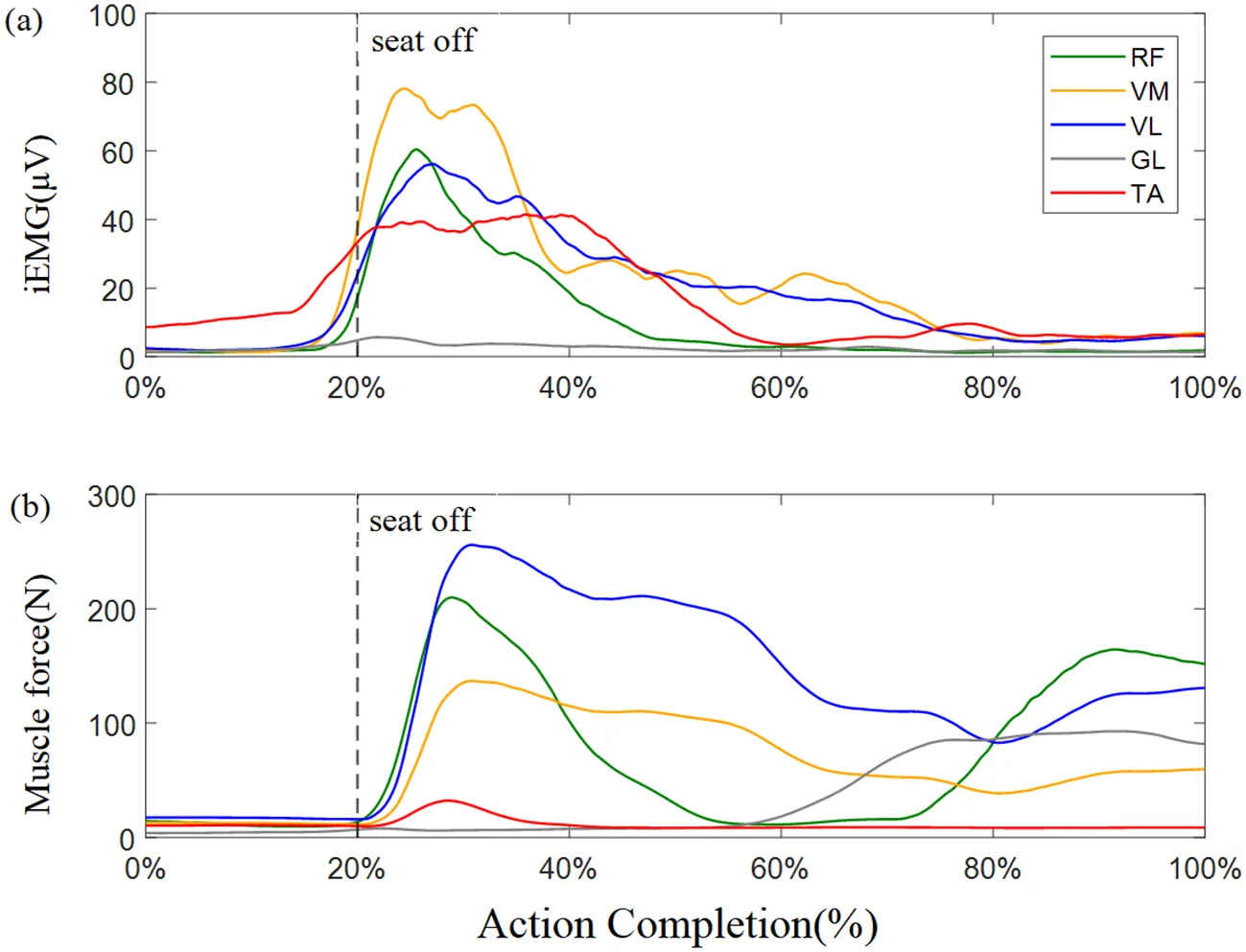
**(A)** EMG signals and **(B)** simulated muscle forces of rectus femoris (RF), vastus medialis (VM), vastus lateralis (VL), gastrocnemius lateralis (GL), and tibialis anterior (TA) during the sit-to-stand task.

Representative time-aligned traces of anterior thigh pressure, seat-off timing, commanded torque, and actuator-output torque are shown in **Figure 9**. The pressure-based trigger occurred before knee-extension onset, after which the controller generated the assigned torque command. Across the 3.0 Nm, 4.5 Nm, and 6.0 Nm conditions, the actuator-output torque followed the commanded torque profile with a finite response delay during the torque-rising and torque-decreasing phases.

**Figure 10:**
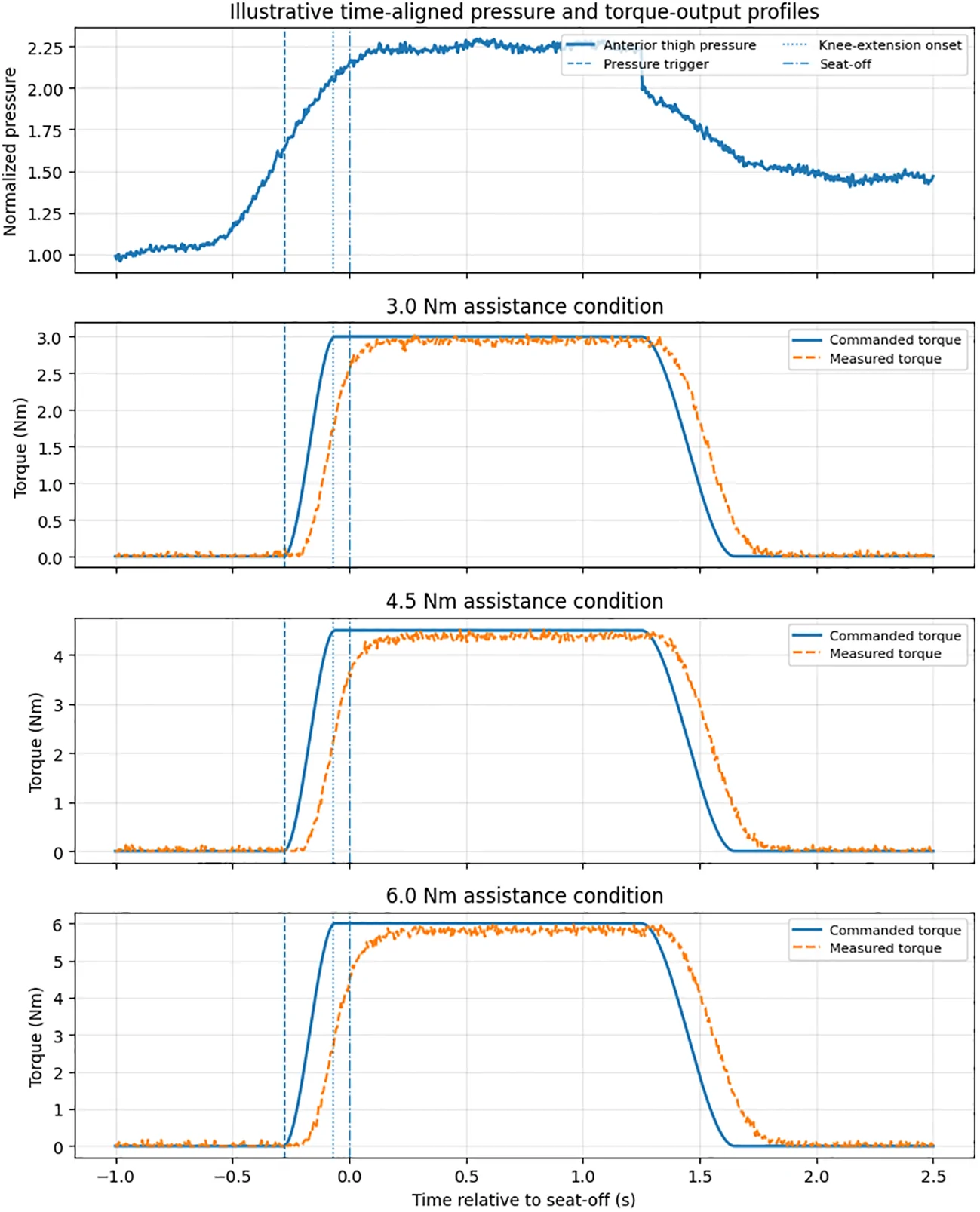
Time-aligned pressure and torque-output profiles during exoskeleton -assisted STS. Representative traces of anterior thigh pressure, pressure-based intention trigger, knee-extension onset, seat-off timing, commanded assistive torque, and measured actuator torque under the 3.0 Nm, 4.5 Nm, and 6.0 Nm assistance conditions. Commanded torque represents the controller output, whereas measured torque represents actuator feedback or motor-current-estimated torque.

The torque-tracking performance metrics are summarized in **Table 1**. The measured peak torque values were close to the commanded torque levels across all assistance conditions. Torque-tracking errors increased with higher commanded torque levels, and the largest errors were mainly observed during the transient torque onset and offset phases rather than during the plateau phase (**Figure 10**).

**Table 1:** Torque-tracking performance under different assistance levels.

Assistance condition	Measured peak torque (Nm)	RMSE (Nm)	MAE (Nm)	Peak absolute error (Nm)
3.0 Nm	3.04 ± 0.08	0.50 ± 0.07	0.29 ± 0.04	1.57 ± 0.21
4.5 Nm	4.53 ± 0.12	0.86 ± 0.11	0.53 ± 0.07	2.72 ± 0.34
6.0 Nm	6.00 ± 0.15	1.25 ± 0.16	0.78 ± 0.10	3.84 ± 0.46

### 3.4 Effects of initial knee joint angle on muscle force

**Figure 11:**
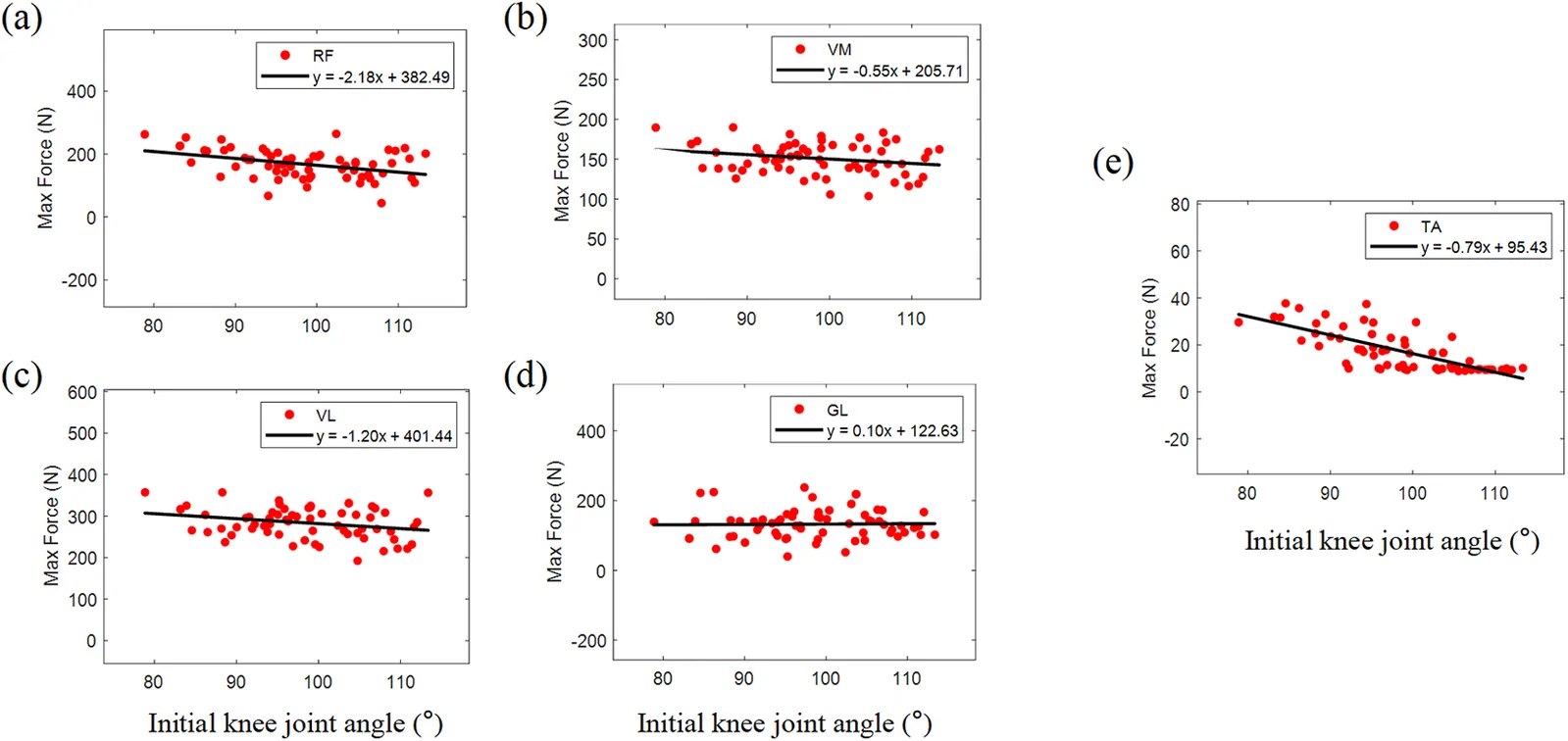
Peak muscle force under different initial knee joint angles with linear regression analysis. Note: the horizontal axis represents the initial knee joint angle (°), and the vertical axis represents peak muscle force (N); the solid black line indicates the linear regression fit; subplots represent **(A)** rectus femoris (RF), **(B)** vastus medialis (VM), **(C)** vastus lateralis (VL), **(D)** gastrocnemius lateralis (GL), and **(E)** tibialis anterior (TA).

Increasing the initial knee joint angle from 80° to 100° was associated with a reduction in quadriceps muscle force during the sit-to-stand task. Peak muscle force of the rectus femoris, vastus medialis, and vastus lateralis decreased progressively with increasing initial angle, whereas the gastrocnemius lateralis showed minimal change across conditions (**Figure 11**). Among these muscles, the rectus femoris demonstrated the greatest sensitivity to angle changes.

**Figure 12:**
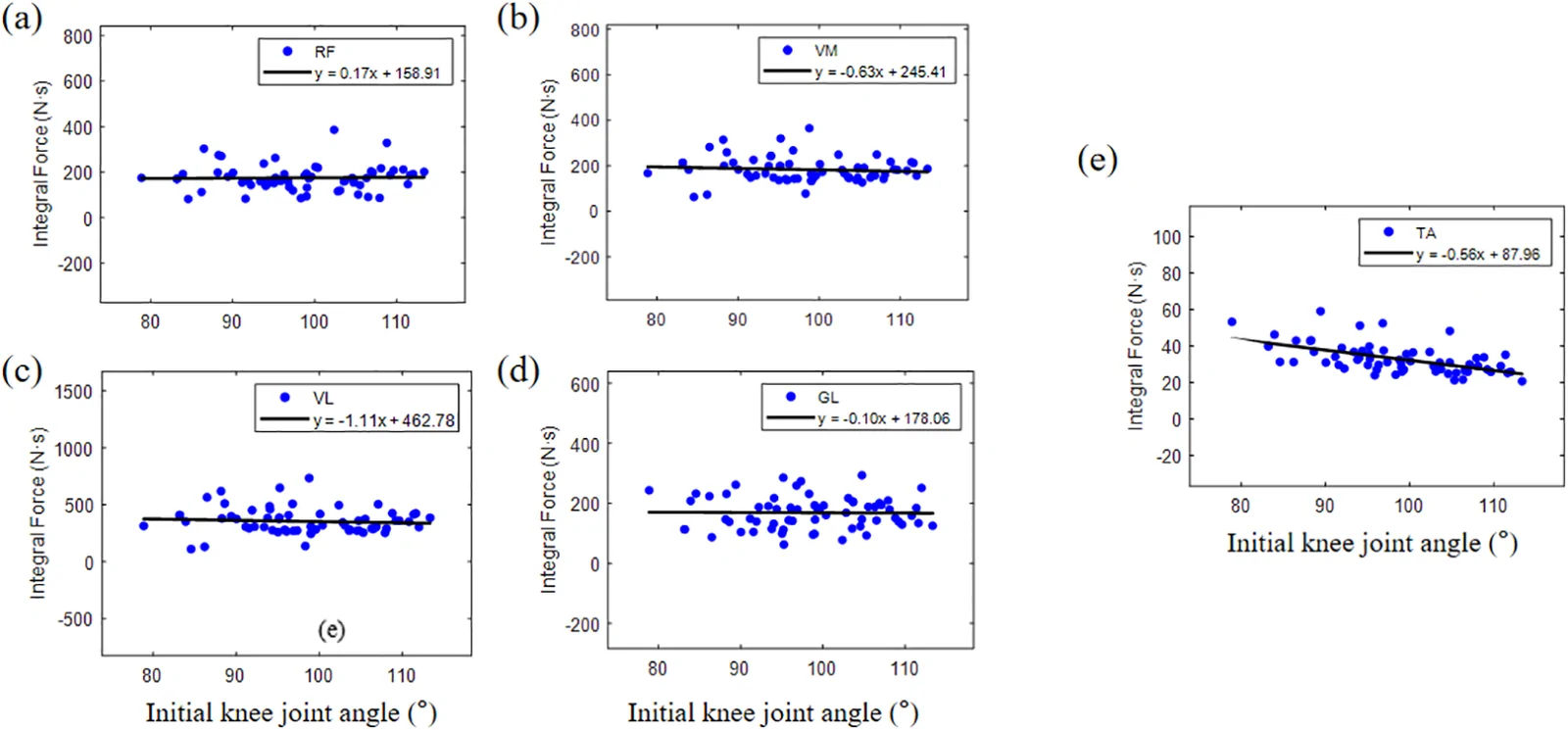
Time-integrated muscle force under different initial knee joint angles with linear regression analysis. Note: the horizontal axis represents the initial knee joint angle (°), and the vertical axis represents time-integrated muscle force (N·s); the solid black line indicates the linear regression fit; subplots represent **(A)** rectus femoris (RF), **(B)** vastus medialis (VM), **(C)** vastus lateralis (VL), **(D)** gastrocnemius lateralis (GL), and **(E)** tibialis anterior (TA).

Time-integrated muscle force showed similar trends. The vastus medialis and vastus lateralis exhibited noticeable reductions across conditions, whereas the rectus femoris and gastrocnemius lateralis showed relatively small changes (**Figure 12**). Overall, increasing the initial knee joint angle reduced quadriceps loading and decreased overall muscular effort during the sit-to-stand task.

### 3.5 Muscle activation and muscle force during the sit-to-stand task

EMG signals increased prior to seat-off (approximately 15% of the movement cycle), whereas muscle forces remained close to zero until seat-off (approximately 20%) (**Figure 9A**). Following seat-off, muscle forces increased rapidly and reached peak values at approximately 30%, with peak forces of 209 aN (RF), 137 aN (VM), 255 aN (VL), and 32 aN (TA) (**Figure 9B**). The vastus lateralis showed the highest force among all muscles. After standing, major muscles maintained moderate force levels for postural stability.

### 3.6 Effects of exoskeleton assistance on knee joint mechanics

**Figure 13:**
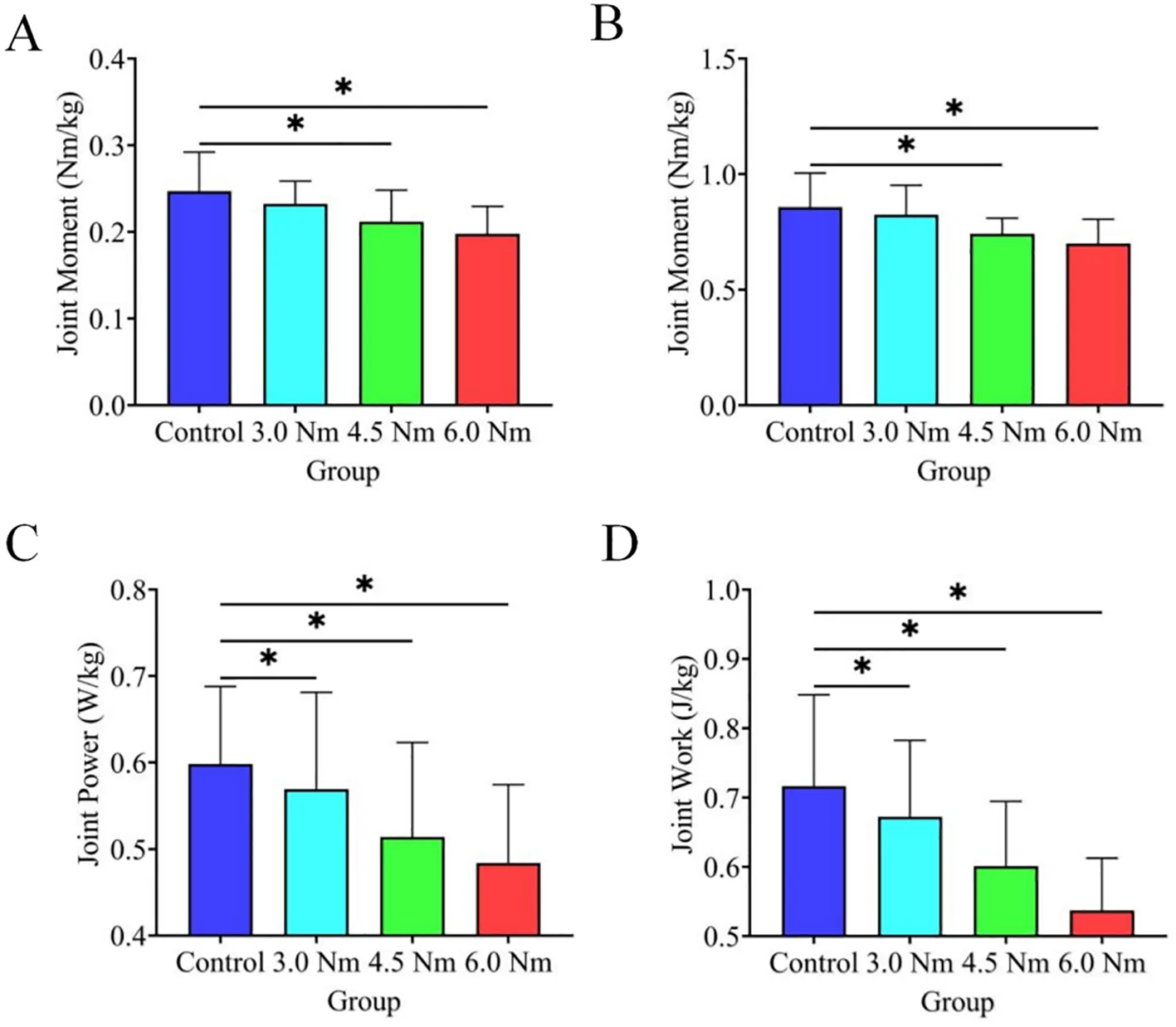
Effects of different exoskeleton assistance levels on knee joint mechanical variables during the sit-to-stand task. Control represents the condition without exoskeleton assistance, and 3.0 Nm, 4.5 Nm, and 6.0 Nm represent different assistance torque levels. Values are normalized to body mass, and error bars represent standard deviation. * indicates p<0.0167 compared with the control condition. Subplots: **(A)** mean joint moment, **(B)** peak joint moment, **(C)** mean joint power, and **(D)** peak joint power.

Knee joint mechanical variables under different assistance conditions are presented in **Figure 13**. For joint moment, mean values decreased from 0.25 ± 0.05 Nm/kg in the control condition to 0.21 ± 0.04 Nm/kg and 0.20 ± 0.03 Nm/kg in the 4.5 Nm and 6.0 Nm conditions, respectively. Both conditions were significantly lower than control (p<0.0167). Peak joint moment showed a similar trend, decreasing from 0.86 ± 0.15 Nm/kg in the control condition to 0.74 ± 0.07 Nm/kg and 0.70 ± 0.11 Nm/kg in the 4.5 Nm and 6.0 Nm conditions (p<0.0167).

For joint power, mean power decreased from 0.18 ± 0.01 W/kg in the control condition to 0.16 ± 0.02 W/kg and 0.15 ± 0.02 W/kg in the 4.5 Nm and 6.0 Nm conditions, respectively (p<0.0167). Peak power decreased from 0.60 ± 0.09 W/kg in the control condition to 0.57 ± 0.11 W/kg, 0.51 ± 0.11 W/kg, and 0.48 ± 0.09 W/kg in the 3.0 Nm, 4.5 Nm, and 6.0 Nm conditions, respectively, with all assisted conditions showing significant reductions compared with control (p<0.0167).

For mechanical work, total joint work decreased from 0.72 ± 0.13 J/kg in the control condition to 0.67 ± 0.11 J/kg, 0.60 ± 0.09 J/kg, and 0.53 ± 0.08 J/kg in the 3.0 Nm, 4.5 Nm, and 6.0 Nm conditions, respectively. All assisted conditions were significantly lower than control (p<0.0167).

## 4 Discussion

### 4.1 Main findings and phase-dependent knee joint moment

The present study demonstrated that the knee joint moment during the sit-to-stand (STS) task exhibits a clear phase-dependent pattern, characterized by a rapid increase after seat-off followed by a gradual decline, with a distinct peak occurring during the early extension phase. This finding supports our initial hypothesis that the STS movement involves a pronounced peak in knee joint moment during the body elevation phase. The sharp rise in joint moment immediately after seat-off indicates that this period represents the highest mechanical demand, during which the knee extensors must generate sufficient torque to overcome gravitational load and initiate vertical displacement of the body.

From a biomechanical perspective, this phase-dependent behavior can be attributed to the combined effects of center-of-mass transition and moment arm variation. After seat-off, the forward and upward movement of the center of mass increases the external moment arm of the ground reaction force relative to the knee joint, thereby requiring a rapid increase in knee extensor torque. As the body approaches an upright posture, the moment arm decreases and the mechanical demand is reduced, resulting in a progressive decline in joint moment. This interpretation is consistent with previous findings showing that peak knee extension moments occur during the rising phase of the STS task (Yoshioka et al., 2014).

In addition, the observed pattern highlights the critical role of knee extensor function in determining STS performance. Previous studies have demonstrated that lower-limb mechanical capacity, particularly knee extensor strength, is a key determinant of successful task execution (Van der Heijden et al., 2009). Furthermore, STS performance is influenced by multiple interacting biomechanical factors, including joint moment requirements and task constraints, emphasizing the complexity of movement control (Janssen et al., 2002). Therefore, analyses focusing solely on kinematic or kinetic variables may not fully capture the underlying control mechanisms of the STS task.

Importantly, the identification of a distinct peak in knee joint moment provides a direct biomechanical basis for assistive strategy design. Since the early extension phase represents the period of highest mechanical demand, appropriately timed assistance during this phase is more likely to effectively reduce the required biological joint moment. This finding establishes a critical temporal window for the application of assistive torque and provides essential guidance for optimizing exoskeleton-assisted STS performance.

### 4.2 Postural and neuromuscular control strategies

The sit-to-stand (STS) movement is widely recognized as a complex functional task that requires not only sufficient mechanical output but also highly coordinated postural and neuromuscular control. The results of the present study, particularly the phase-dependent increase in knee joint moment, should therefore be interpreted within the broader framework of whole-body coordination and neural control strategies. Previous research has consistently demonstrated that STS is a multi-phase movement involving the integration of anticipatory postural adjustments, coordinated muscle activation, and dynamic balance regulation (Schenkman et al., 1990; Janssen et al., 2002).

From a postural control perspective, the initiation of STS is characterized by a forward displacement of the center of mass (COM), which occurs prior to seat-off. This anticipatory adjustment facilitates the transition from a stable seated posture to dynamic movement and reduces the mechanical demand of the subsequent extension phase (Mourey et al., 1998). However, this forward shift also introduces instability, requiring precise control to maintain balance. Studies have shown that the central nervous system actively regulates trunk inclination and lower-limb coordination to ensure that the COM remains within the base of support during this transition (Pai and Rogers, 1990). This highlights that STS is not merely a strength-dependent task but also a balance-critical movement.

In addition to postural adjustments, neuromuscular control plays a fundamental role in coordinating joint motion and force production. Electromyographic studies have demonstrated that muscle activation begins prior to the mechanical onset of movement, indicating the presence of feedforward control mechanisms (Goulart and Valls-Solé, 1999; Roldán Jiménez et al., 2019). Specifically, early activation of the tibialis anterior contributes to forward momentum generation, followed by strong activation of the quadriceps during the extension phase. This sequential activation pattern ensures smooth force transmission and efficient movement execution.

The findings of the present study, showing a rapid increase in knee joint moment immediately after seat-off, are consistent with these neuromuscular control strategies. The early and substantial activation of the quadriceps is necessary to generate sufficient knee extension torque to overcome gravitational load. Previous work has confirmed that peak knee joint moments occur during the rising phase of STS, reflecting the high mechanical demand of body elevation (Yoshioka et al., 2014). Moreover, the magnitude of joint moments is closely linked to movement kinematics, indicating that neuromuscular control strategies directly influence mechanical output (Yoshioka et al., 2007).

The earlier increase in EMG activity compared with simulated muscle force reflects the difference between neuromuscular activation and mechanical force production. EMG represents the electrical activation of muscles and anticipatory preparation before movement, whereas the simulated muscle forces are determined by the mechanical demands of the task and increase markedly only after seat-off, when chair support is removed and the knee extensors must elevate the body. This temporal pattern supports the rationale for pressure-based intention recognition: early quadriceps activation may produce local soft-tissue expansion under the anterior thigh cuff before clear knee extension occurs, allowing the pressure sensor to detect movement intention earlier than purely kinematic criteria. However, anterior thigh pressure should not be interpreted as a direct substitute for EMG, and further work is required to quantify the relationship among EMG activation, cuff pressure changes, and muscle force.

Another critical aspect of neuromuscular control during the STS task is the dynamic coordination among multiple joints. Rather than acting independently, the hip, knee, and ankle joints function as a coupled system, enabling efficient control of movement trajectory and mechanical demand. This coordinated behavior ensures smooth transfer of mechanical energy across body segments while preventing excessive loading at any single joint. Experimental studies have demonstrated that STS movement involves tightly coordinated inter-joint patterns, particularly between the hip-knee and knee-ankle pairs, reflecting an integrated motor control strategy (Papa and Cappozzo, 2000; He et al., 2023). Disruptions in this coordination may lead to inefficient movement patterns and increased joint loading.

From a biomechanical perspective, inter-joint coordination is closely associated with the regulation of movement variability and adaptability. Recent evidence suggests that coordination patterns are not rigid but exhibit controlled variability, which allows the neuromuscular system to adapt to changing task demands or fatigue conditions (Chen and Chou, 2022). This variability reflects a flexible control strategy in which joint contributions are continuously adjusted to maintain performance stability despite internal or external perturbations.

The efficiency of these control strategies is strongly influenced by individual functional capacity. In older adults and individuals with neuromuscular impairments, altered muscle activation patterns and delayed recruitment timing are commonly observed, leading to reduced movement efficiency and increased reliance on compensatory strategies (Janssen et al., 2002). For example, increased trunk flexion is often used to reduce knee joint loading, but this may compromise postural stability and increase fall risk.

In the later phase of the STS task, as the body approaches an upright posture, the primary challenge shifts from generating upward propulsion to achieving stable postural regulation. Recent evidence indicates that the raising and especially the stabilization phase are highly sensitive to balance-related deficits, with older adults exhibiting substantially greater postural sway during these phases, suggesting that control demands remain high even after the major extensor torque requirement has declined. A recent systematic review further showed that balance regulation during STS in older adults is characterized by greater postural sway, increased trunk flexion, and higher agonist-antagonist co-activation at the knee and ankle, indicating a stronger reliance on feedback-mediated neuromuscular regulation as the movement approaches completion. Taken together, these findings support the view that the late phase of STS is dominated less by gross mechanical output and more by fine postural adjustment, sensory integration, and stabilization of the center of mass (Alcazar et al., 2018).

These phase-dependent control characteristics have direct implications for exoskeleton control design. Because the mechanical and neuromuscular demands of movement vary across phases, assistive torque should be delivered in a way that supports the high-demand rising phase without interfering with the subsequent stabilization phase. Recent work on wearable robots has emphasized that high-performance assistance depends on close integration between the device and human motor control, rather than simply increasing actuator output. In parallel, studies on human-exoskeleton interaction have shown that assistance becomes more effective when it is coordinated with the user’s movement timing and neuromuscular behavior, highlighting the importance of temporal compatibility in control strategies. Therefore, the control strategy used in the present study appears advantageous because it targets assistance to the mechanically demanding portion of STS while avoiding unnecessary disturbance during the late stabilization phase. In this sense, the effectiveness of our controller is not only a matter of torque magnitude, but also of phase-specific matching between assistive output and the user’s natural postural and neuromuscular regulation (Mohebbi, 2020; Diaz et al., 2022).

### 4.3 Effects of exoskeleton assistance

The present study demonstrated that exoskeleton assistance significantly reduced knee joint mechanical demand during the STS task, as evidenced by consistent decreases in joint moment, power, and mechanical work across assisted conditions. These findings support the hypothesis that externally applied assistive torque can effectively substitute part of the biological joint loading required to perform the movement.

From a joint moment perspective, both mean and peak knee joint moments were significantly reduced under the 4.5 Nm and 6.0 Nm conditions compared with the control condition. This reduction indicates that the exoskeleton provided effective mechanical support during the extension phase of STS, thereby decreasing the net torque generated by the musculoskeletal system. Such findings are consistent with the concept of mechanical load redistribution, where external assistance partially replaces internal joint torque requirements. Previous studies have shown that external devices can alter joint loading patterns by redistributing mechanical demand across the human-device system (DeVita and Hortobagyi, 2000; Kao et al., 2010). In the context of STS, where the knee joint plays a dominant role in generating extensor torque to elevate the body, the observed reductions suggest that the applied assistance effectively alleviated the primary mechanical burden associated with vertical body lifting.

In addition to joint moment, reductions in both mean and peak joint power further indicate that exoskeleton assistance altered the energetic requirements of the movement. Joint power reflects the rate of mechanical energy generation, and decreases in power output suggest a reduced demand for active energy production by the lower-limb musculature. This interpretation aligns with previous findings demonstrating that powered exoskeletons can reduce biological energy expenditure by supplementing mechanical work during locomotor tasks (Sawicki and Ferris, 2008; Galle et al., 2017). Although metabolic data were not directly measured in the present study, the observed reductions in joint power imply a potential unloading effect at the neuromuscular level, whereby muscles may require less activation to achieve the same functional outcome.

Similarly, the decrease in total joint mechanical work across all assisted conditions provides further evidence of reduced overall mechanical demand during STS. Mechanical work represents the cumulative energy required to complete the movement, and its reduction suggests that the exoskeleton contributed directly to the generation of movement-related energy. This finding is in agreement with previous research demonstrating that assistive devices can lower the total mechanical and metabolic cost of movement by supplementing biological work output (Mooney et al; Collins et al., 2015). In functional tasks such as STS, which require substantial mechanical output within a short time window, reducing total joint work may have important implications for individuals with limited strength or impaired motor function.

An important observation in the present study is the dose-dependent effect of assistive torque magnitude. While both 4.5 Nm and 6.0 Nm conditions significantly reduced joint moment and power compared with the control condition, the incremental benefits between assistance levels were relatively modest. This suggests that increasing assistive torque does not necessarily lead to proportional reductions in mechanical demand. Such a non-linear relationship between assistance magnitude and biomechanical outcome has been reported in previous studies, where optimal assistance levels were found to maximize performance benefits without disrupting natural movement patterns (Zhang et al., 2017; Ding et al., 2018). Excessive assistance may potentially interfere with intrinsic motor control strategies, leading to diminished coordination efficiency or altered movement patterns.

Taken together, these findings highlight that effective exoskeleton assistance is not solely dependent on the magnitude of torque output, but also on its alignment with the temporal and mechanical demands of the task. The reductions in joint moment, power, and work observed in the present study suggest that the implemented assistance strategy achieved a degree of mechanical substitution while maintaining functional movement execution. This supports the broader principle that successful human-exoskeleton interaction requires both magnitude-appropriate and task-specific assistance to ensure compatibility with the user’s natural motor control system.leton assistance.

The torque-tracking analysis further confirmed that the actuator output generally followed the commanded assistance levels. However, transient tracking errors were observed during torque onset and offset, reflecting the finite response time of the motor-control system. Therefore, the biomechanical effects observed in the present study should be interpreted in relation to the actual actuator-output profile rather than the nominal peak torque alone.

It should be noted that the present assisted-condition analysis focused primarily on knee-joint mechanical outcomes. Although participants completed a familiarization session, adopted a standardized initial posture, and performed the STS task at a self-selected natural speed, we did not conduct a comprehensive quantitative comparison of whole-body movement strategy across assistance conditions. Specifically, movement duration, trunk inclination, hip joint moment, and ankle joint moment were not statistically analyzed in the present study. Therefore, the observed reductions in knee joint moment, power, and mechanical work should be interpreted as evidence of reduced knee-joint mechanical demand under the tested conditions, rather than as proof that the overall movement strategy remained unchanged. Future studies should include whole-body kinematic and kinetic analyses to determine whether knee assistance induces compensatory changes at the trunk, hip, or ankle.

### 4.4 Implications

A key contribution of this study lies in the development and validation of a pressure-based motion intention recognition strategy, which demonstrated improved temporal alignment between assistive torque output and the user’s movement initiation.

Compared with conventional kinematics-based approaches, which are limited by system delays and signal processing latency, the proposed pressure-threshold method enabled earlier detection of movement onset. Specifically, the assistive torque was initiated with a delay of only 0.072 s relative to the onset of knee joint motion, representing a substantial improvement in responsiveness. This enhanced temporal synchronization is critical for ensuring effective human-exoskeleton interaction, as timely assistance allows the device to support the user during the most mechanically demanding phase of the movement. From a control perspective, this approach provides a feasible and practical solution for real-time intention detection without relying on complex biosignals such as electromyography, thereby improving system portability and usability.

The observed reductions in knee joint moment, power, and mechanical work suggest that the proposed assistance strategy can reduce lower-limb mechanical demand during a standardized STS task in healthy young participants. These findings provide biomechanical evidence that may inform the future design of assistive strategies for populations with reduced strength or impaired motor function. However, because older adults and patients may exhibit different neuromuscular control, balance regulation, and movement strategies, the present results should not be interpreted as direct evidence of clinical efficacy. Future studies involving target user populations are required to determine whether similar unloading effects can be achieved in rehabilitation or daily-life contexts.

### 4.5 Limitations

Several limitations should be considered when interpreting the present findings. The participant sample consisted exclusively of healthy young male individuals, which may limit the generalizability of the results to other populations. Movement strategies, neuromuscular capacity, and joint loading characteristics can differ substantially in older adults or individuals with functional impairments, and these differences may influence both the effectiveness of intention recognition and the biomechanical response to exoskeleton assistance. Therefore, the present findings should be regarded as laboratory-based biomechanical evidence in healthy participants and should not be directly generalized to older adults, patients requiring assistance, or clinical rehabilitation settings without further validation.

The analysis focused on kinematic and kinetic variables without direct measurement of muscle activation. As a result, conclusions regarding neuromuscular unloading remain indirect and should be interpreted with caution. Although reductions in joint moment, power, and mechanical work suggest a decreased mechanical demand, the underlying changes in muscle coordination and activation patterns cannot be fully determined without electromyographic data.

All experiments were conducted under controlled laboratory conditions using a standardized sit-to-stand task. While this approach ensured high repeatability and internal validity, it may not fully reflect the variability encountered in real-world environments. Factors such as movement speed, fatigue, and environmental constraints may alter both motor control strategies and the interaction between the user and the exoskeleton, which could influence the applicability of the proposed approach in practical settings.

The assistive torque levels were predefined and applied uniformly across participants, without accounting for individual differences in strength, body mass, or movement patterns. Since optimal assistance is likely to vary between individuals and tasks, the use of fixed torque levels may limit the overall effectiveness of the system. Adaptive or individualized assistance strategies may provide improved performance and should be explored in future work.

In addition, the evaluation of intention recognition performance was conducted within the specific context of the present experimental setup. Although the pressure-based method demonstrated improved temporal responsiveness, its robustness under different wearing conditions, sensor placements, or long-term use remains to be further validated.

Finally, although the pressure-based method achieved successful positive-event detection in all validation recordings, the present validation protocol did not include non-intention events, quiet sitting, posture adjustments, or other negative-control movements. Therefore, specificity, false-positive rate, and overall classification accuracy could not be determined. Future studies should include both positive STS trials and negative non-intention trials to comprehensively evaluate the diagnostic performance and robustness of the recognition algorithm.

## 5 Conclusion

This study systematically characterized the phase-dependent knee joint mechanical demand during the sit-to-stand task and evaluated the effectiveness of a knee exoskeleton assistance strategy based on motion intention recognition. The results demonstrated that knee joint moment exhibits a distinct temporal pattern, with peak demand occurring during the body elevation phase, highlighting the critical role of the knee joint in overcoming gravitational loading. The pressure-based intention recognition method enabled earlier detection of movement onset, effectively compensating for system delays and improving the temporal alignment between assistive torque and user movement. This improved synchronization allowed the exoskeleton to provide support during the most mechanically demanding phase of the task. Exoskeleton assistance significantly reduced knee joint moment, power, and mechanical work, indicating effective reduction of mechanical loading on the lower-limb musculoskeletal system. These findings suggest that assistance aligned with both the magnitude and timing of knee joint mechanical demand can improve human-exoskeleton interaction during a standardized STS task. Overall, the integration of early intention recognition with phase-dependent assistive torque provides a feasible framework for optimizing assistive strategies in functional movements.

Overall, the integration of early intention recognition with phase-dependent assistive torque provides a feasible framework for optimizing assistive strategies during standardized functional movements in healthy participants. Its applicability to rehabilitation, mobility assistance, or clinical populations requires further investigation in older adults, patients with functional impairments, and real-world environments.

## Data Availability

The raw data supporting the conclusions of this article will be made available by the authors, without undue reservation.
