# Supplementary material for: A pressure-based motion intention recognition strategy for phase-specific knee exoskeleton assistance during sit-to-stand
Source: Front Physiol. 2026 Sep 4;17:1859056. doi: 10.3389/fphys.2026.1859056 (PMC13586795; doi:10.3389/fphys.2026.1859056)
Supplement: Supplementary file 1 [file Supplementaryfile1.docx]

# Human-Machine Interaction Software

The data communication program deployed on the embedded control board enables real-time transmission of sensor data to a host PC via serial communication. Although the transmitted data are parsed at the software level, they are not sufficiently intuitive for clinical users such as physicians or rehabilitation therapists. Therefore, a user-friendly human–machine interaction (HMI) interface is essential for monitoring and controlling the knee exoskeleton during rehabilitation training. Through a graphical user interface (GUI), operators can visualize real-time movement information of the user, evaluate motion performance and accuracy, and precisely control knee joint movement.

The HMI interface was developed using the Qt 5.12 application framework, as illustrated in Figure 2.17.

Figure 2.17. Human–machine interaction software interface.

The “Serial Communication” module is responsible for establishing communication between the host PC and the embedded control board. After launching the software, a baud rate of 115200 is selected, and the “Open Port” function automatically detects and connects to the corresponding COM port. The communication can be terminated by selecting “Close Port,” while the “Refresh” function allows port re-detection, and the “Clear” function removes data from the buffer.

Incoming data from the control board are stored in the serial buffer and read sequentially by the program. Based on the predefined communication protocol, the data are decoded and dynamically displayed on the interface. The GUI includes three real-time plotting panels: IMU data, pressure data, and motor information.

The IMU data panel contains three tabs corresponding to acceleration, angular velocity, and Euler angles. Each tab displays three-axis data in real time, allowing synchronized visualization of motion signals.

The pressure data panel simultaneously displays data from four sensor channels within a single plotting area, enabling monitoring of pressure distribution across different attachment locations.

The motor information panel includes three tabs for velocity, position, and current, each presenting real-time curves of the corresponding motor parameters.

The “Motor Control” module provides real-time status feedback, such as speed mode and position mode, and allows control of motor operation through commands including “Start,” “Stop,” and “Standby.”

The “Joint Control” module includes four control modes: speed mode, position mode, step mode, and torque mode. In speed mode, the required inputs are angular velocity (rad/s) and movement direction (flexion/extension). In position mode, inputs include angular velocity (rad/s) and target position (deg). In step mode, inputs include angular velocity (rad/s), step angle (deg), and direction (flexion/extension). In torque mode, the required inputs are torque magnitude (Nm) and direction (flexion/extension). After selecting the control mode and parameters, commands are transmitted to the embedded system via serial communication by clicking “Send.” The “Reset” function clears all input parameters, while the “Home” function returns the exoskeleton to the neutral (0°) position.

In addition to these functions, the HMI software supports advanced features such as complex motion control and motion intention recognition.
