# Supplementary material for: A pressure-based motion intention recognition strategy for phase-specific knee exoskeleton assistance during sit-to-stand
Source: Front Physiol. 2026 Sep 4;17:1859056. doi: 10.3389/fphys.2026.1859056 (PMC13586795; doi:10.3389/fphys.2026.1859056)
Supplement: Supplementary file 2 [file Supplementaryfile2.docx]

# Motion Intent Recognition

Motion intention recognition refers to identifying and predicting a user’s intended movement through technical approaches. In the sit-to-stand (STS) task, knee extension begins at the moment when the body leaves the seat. The knee joint moment–angle relationship (Figure 1) shows that joint moment rapidly increases to its peak within approximately 5° after the onset of knee extension. Therefore, the key to intention recognition lies in accurately detecting the seat-off event, followed by rapid activation of exoskeleton assistance.

The exoskeleton system can acquire knee joint angle and angular velocity in real time using embedded encoders. A straightforward approach is to detect the onset of knee extension based on kinematic signals and then apply assistive torque. However, due to delays associated with command transmission, communication, and motor actuation, a latency of approximately 0.3 s exists between intention detection and torque output. As a result, motion intention recognition based solely on kinematic data is not sufficiently reliable for real-time control.

Figure 1.Changes in knee joint velocity and pressure data during the sitting-up movement

The quadriceps muscle is the primary driver of knee extension. Prior to joint movement, muscle fibers generate electrical activity and produce force, initiating limb motion. Although electromyography (EMG) signals can predict movement intention before observable motion occurs, their application in wearable exoskeleton systems is limited due to complex setup requirements and sensitivity to environmental conditions. In contrast, pressure sensors placed at the anterior thigh, between the quadriceps and soft padding, can capture muscle expansion during contraction. As shown in Figure 2, pressure signals at the anterior thigh exhibit more pronounced changes than those at other locations. The pressure reaches its peak at the onset of knee extension, and the increase in pressure precedes the onset of positive angular velocity by approximately 0.5 s. Therefore, pressure-based signals provide a more practical and effective approach for predicting knee joint motion.

Figure 2.Pressure changes on the anterior thigh during sit-up movements in 13 subjects

Figure 2 illustrates the pressure profiles of 13 participants during the STS task. Although differences in limb morphology, clothing thickness, and attachment tightness result in variability in baseline pressure, peak values, and rates of change, all curves exhibit a consistent trend characterized by an increase from baseline to peak followed by a return to baseline. Since the peak pressure corresponds to the onset of knee extension, identifying an intermediate feature point within this rising phase enables early prediction of movement initiation.

Between the onset and peak of the pressure signal, the rate of change exhibits a characteristic pattern in which it initially increases and then decreases, suggesting the presence of an inflection point. Conventional approaches, such as smoothing followed by derivative-based peak detection or polynomial fitting, can identify such inflection points under offline conditions. However, these methods are not suitable for real-time applications, as continuously updated data may alter the detected inflection point, making them impractical for online exoskeleton control.

These approaches do not account for baseline pressure variations. As shown in Figure 2, when baseline pressure is lower, the peak pressure is correspondingly reduced, indicating that the overall magnitude of the pressure signal is proportional to the baseline level. This relationship arises because sensor output is proportional to input force, while variations in attachment tightness alter the contact conditions between the sensor and the body. Tighter attachment leads to amplified pressure signals, whereas looser attachment reduces signal magnitude.

Figure 3 presents pressure data from a representative participant, where the baseline pressure was 1450 g and the peak pressure reached 4080 g. The onset point (A), peak point (E), and intermediate points (B, C, D), corresponding to 25%, 50%, and 75% of the horizontal distance between A and E, were identified. By analyzing these characteristic points across all participants, the ratios of pressure values at B, C, D, and E relative to the baseline were calculated, as summarized in Table 1.

Figure 3. Initial point (A), peak point (E), and quantile points (B, C, D)

Table 1.Relationship between pressure values at different quantile points and resting values: YX represents the pressure value at point X (g)

| subjects | *Y_A_* | *Y_E_* | *Y_B_* / *Y_A_* | *Y_C_* / *Y_A_* | *Y_D_* / *Y_A_* | *Y_E_* / *Y_A_* |
| --- | --- | --- | --- | --- | --- | --- |
| 1 | 650 | 1840 | 1.44 | 1.88 | 2.34 | 2.83 |
| 2 | 615 | 1732 | 1.59 | 1.90 | 2.47 | 2.82 |
| 3 | 674 | 1952 | 1.43 | 1.88 | 2.26 | 2.90 |
| 4 | 1237 | 3350 | 1.45 | 1.87 | 2.38 | 2.71 |
| 5 | 1190 | 3512 | 1.53 | 2.00 | 2.45 | 2.95 |
| 6 | 1450 | 4080 | 1.49 | 1.82 | 2.30 | 2.80 |
| 7 | 1150 | 3308 | 1.33 | 1.85 | 2.35 | 2.88 |
| 8 | 1360 | 3970 | 1.41 | 1.85 | 2.42 | 2.92 |
| 9 | 753 | 2620 | 1.41 | 2.19 | 2.79 | 3.48 |
| 10 | 892 | 2832 | 1.41 | 1.88 | 2.46 | 3.07 |
| 11 | 1050 | 3194 | 1.22 | 1.82 | 2.31 | 3.04 |
| 12 | 675 | 1930 | 1.21 | 1.81 | 2.31 | 2.86 |
| 13 | 861 | 2395 | 1.46 | 1.76 | 2.18 | 2.78 |
| average | / | / | 1.41 | 1.89 | 2.38 | 2.93 |

Table 1 shows that the pressure values at the 25%, 50%, 75%, and peak points are approximately 1.41, 1.89, 2.38, and 2.93 times the baseline pressure, respectively. This indicates that, in real-time control, a threshold coefficient (C) based on baseline pressure can be used to estimate the relative position of the signal between the onset and peak. Based on this observation, a threshold-based method was developed to identify an intermediate point for motion intention prediction.

As illustrated in Figure 4, red circles represent raw pressure data points, black triangles denote the average value calculated over every 10 data points, and pink squares indicate the identified intermediate point. The pressure data are continuously updated in real time. For every 10 newly acquired data points, the average value is calculated and denoted as average1, which is plotted as a black triangle.

Once five average1 values have been accumulated, a second-level average, denoted as average2, is computed from these five values. If fewer than five average1 values are available, the first average1 is used as average2.

When the sixth average1 is obtained, the following condition is evaluated:

If this condition is satisfied, it indicates that the pressure signal has begun to rise, and this moment is defined as the onset of the sit-to-stand movement. Otherwise, average2 is recalculated using the most recent five average1 values until the condition is satisfied.

After the onset point has been identified, the incoming pressure data continue to be updated. If the magnitude of a data point satisfies the following condition:

This data point is identified as the target point. The corresponding value in the data sequence is then defined as the intermediate point.

Figure 4. Threshold method for locating intermediate points

Figure 5 presents an example of the threshold-based method for identifying the intermediate point. For the first 50 data samples, the corresponding average1 values were 871.7, 874.5, 868.5, 861.3, and 879.5, resulting in an average2 value of 871.1.

When the number of accumulated data points reached 60, the sixth average1 was calculated as 884.8. Since 884.8 < 871.1 + 50, the condition was not satisfied, and the iteration continued. The value of average2 was then updated to 873.7 based on the most recent five average1 values. Upon further iteration, the next average1 reached 960.3, satisfying the condition and thereby identifying the onset point of the movement.

Subsequently, the data continued to update until the magnitude of a data point reached 1638, which satisfied the predefined condition and was thus identified as the intermediate point.

To evaluate the effectiveness of the proposed algorithm, an additional experiment was conducted with six participants wearing the exoskeleton system. The intermediate points identified from the six sets of pressure data are shown in Figure 5. These points corresponded to the 0.52, 0.51, 0.55, 0.50, 0.44, and 0.42 quantiles of each dataset, with a mean value of 0.49, demonstrating the reliability and consistency of the threshold-based method for intermediate point detection.

Figure 5. Identification of midpoint values for six datasets using the threshold method (C = 1.89)

The threshold-based algorithm was implemented in the exoskeleton control system, and the results are presented in Figure 6. The onset of assistive torque output exhibited a delay of only 0.072 s relative to the onset of knee joint motion. Compared with the approach based solely on kinematic data, the proposed method advanced the timing of torque output by approximately 0.228 s, thereby demonstrating its effectiveness in motion intention recognition.

Figure 6.Effectiveness of identifying movement intent using the pressure data threshold method
